# Impact of Degree of Ionization and PEGylation on the Stability of Nanoparticles of Chitosan Derivatives at Physiological Conditions

**DOI:** 10.3390/md20080476

**Published:** 2022-07-25

**Authors:** André Miguel Martinez Junior, Aline Margarete Furuyama Lima, Grazieli Olinda Martins, Vera Aparecida de Oliveira Tiera, Mohamed Benderdour, Julio Cesar Fernandes, Marcio José Tiera

**Affiliations:** 1 Department of Chemistry and Environmental Sciences, IBILCE, São Paulo State University—UNESP, São José do Rio Preto 15054-000, SP, Brazil; andre.martinez@unesp.br (A.M.M.J.); aline.margarete@nanovetores.com.br (A.M.F.L.); grazieli.martins@unesp.br (G.O.M.); vera.oliveira-tiera@unesp.br (V.A.d.O.T.); 2 Orthopedic Research Laboratory, Hôpital du Sacré-Coeur de Montréal, Université de Montréal-Canada, Montreal, QC H3C3J7, Canada; mohamed.benderdour@umontreal.ca (M.B.); julio.c.fernandes@umontreal.ca (J.C.F.)

**Keywords:** gene therapy, siRNA, nanoparticles, DIPEA, physiological pH

## Abstract

Nowadays, the therapeutic efficiency of small interfering RNAs (siRNA) is still limited by the efficiency of gene therapy vectors capable of carrying them inside the target cells. In this study, siRNA nanocarriers based on low molecular weight chitosan grafted with increasing proportions (5 to 55%) of diisopropylethylamine (DIPEA) groups were developed, which allowed precise control of the degree of ionization of the polycations at pH 7.4. This approach made obtaining siRNA nanocarriers with small sizes (100–200 nm), positive surface charge and enhanced colloidal stability (up to 24 h) at physiological conditions of pH (7.4) and ionic strength (150 mmol L^−1^) possible. Moreover, the PEGylation improved the stability of the nanoparticles, which maintained their colloidal stability and nanometric sizes even in an albumin-containing medium. The chitosan-derivatives displayed non-cytotoxic effects in both fibroblasts (NIH/3T3) and macrophages (RAW 264.7) at high N/P ratios and polymer concentrations (up to 0.5 g L^−1^). Confocal microscopy showed a successful uptake of nanocarriers by RAW 264.7 macrophages and a promising ability to silence green fluorescent protein (GFP) in HeLa cells. These results were confirmed by a high level of tumor necrosis factor-α (TNFα) knockdown (higher than 60%) in LPS-stimulated macrophages treated with the siRNA-loaded nanoparticles even in the FBS-containing medium, findings that reveal a good correlation between the degree of ionization of the polycations and the physicochemical properties of nanocarriers. Overall, this study provides an approach to enhance siRNA condensation by chitosan-based carriers and highlights the potential of these nanocarriers for in vivo studies.

## 1. Introduction

Over the last decade, advances in RNA engineering have enabled the obtaining of small interfering RNAs (siRNA) with enhanced pharmacologic activities, which has driven an increased interest in establishing therapeutic platforms for the treatment of several diseases [1]. Despite improvements, the therapeutic efficiency of siRNA is still limited by the use of efficient vectors capable of directing them to their site of action, inside the target cells [2]. In this scenario, the use of non-viral vectors has gained prominence, especially after the use of lipid nanoparticles in mRNA vaccines applied in the fight against the Coronavirus Disease 2019 (COVID-19) pandemic [3] and for the transport of siRNA in the first RNA interference (RNAi) drug approved by the US Food and Drug Administration (FDA) in 2018 [2].

In this context, chitosan has emerged as a non-toxic, highly biocompatible, biodegradable, and low-cost polycation that is able to transport nucleic acids to target cells through the formation of nanocarriers [4,5]. Recent studies showed that siRNA/chitosan nanoparticles may be promising candidates for the treatment of several disorders, for example: HIV infection [6], bladder cancer [7], Huntington’s disease [8], and tuberculosis [9]. However, due to the low pKa (6.2–6.4) of its amine groups, the application of chitosan nanoparticles may be limited in neutral/basic pH environments, such as in the blood (pH 7.4), eye mucous (pH 7.8), and jejunum (pH 7–8) [4,10,11]. In order to overcome this limitation, some research groups have been modifying chitosan with additional amino groups of higher pKa to increase its applicability at neutral pH and the results have shown more compact, stable, and efficient siRNA nanocarriers [12,13,14,15]. Particularly, the grafting of hydrophobic-ionizable groups containing tertiary amines, such as diethylaminoethyl (DEAE) and diisopropylethylamine (DIPEA) groups that improve the ability of chitosan as a gene therapy agent [15,16,17], is highlighted.

Additionally, the efficiency of chitosan as a transfection agent is also highly dependent on molecular weight (MW) and degree of deacetylation (DDA) [18]. It has been reported that for the formation of stable siRNA/chitosan nanocarriers, polysaccharides 5–10 times larger than siRNA are more appropriate, which correlates with an optimal MW in the range of 65 to 170 kDa [5]. Additionally, Alameh and coworkers demonstrated that chitosans having DDA higher than 92% are superior siRNA delivery systems compared to partially acetylated ones, a result that can be attributed to the higher positive surface charge of the nanoparticles based on highly deacetylated chitosans [18]. In addition to the charge density, PEGylation may avoid destructive electrostatic interactions between these nanocarriers and the negatively charged proteins, favoring the preservation of their structural integrity and increasing their circulation lifetime [10]. It has also been reported that deacetylated chitosan grafted with 2 kDa polyethylene glycol (PEG) led to non-cytotoxic siRNA nanocarriers with enhanced stability and knockdown performance [19]. Moreover, the same positive effect was observed when a low density of PEG chains (2 kDa) was grafted onto polyethyleneimine (PEI) and applied to the siRNA delivery [20].

**Figure 1:**
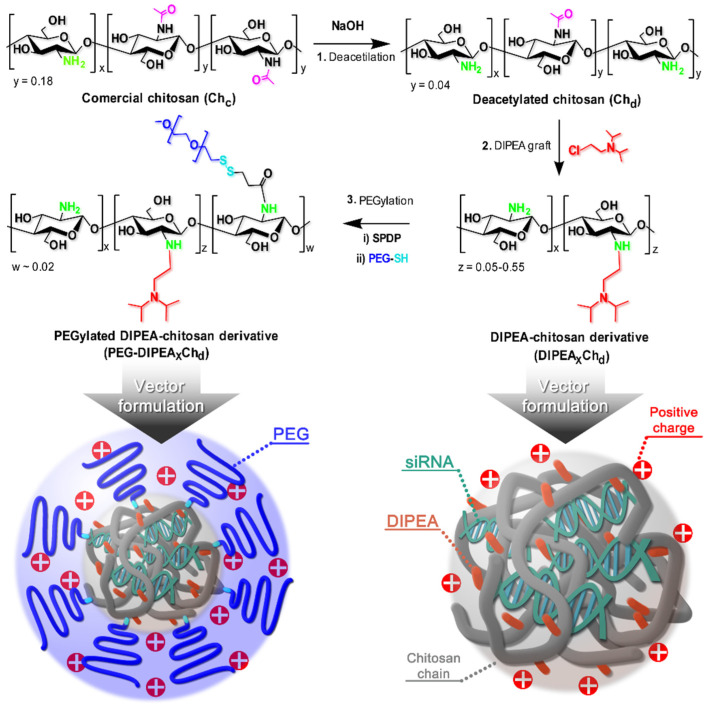
Schematic representation of the synthetic route adopted to obtain chitosan diisopropylethylamine/polyethylene glycol (DIPEA/PEG) derivatives and a symbolic representation of the nanocarriers developed in the study through polymer/siRNA simple complexation.

Recently, our research team has shown that the combination of chitosan with DIPEA groups is a promising alternative for the development of siRNA nanocarriers. However, the best transfection efficiencies were still centered at low pH values due to the higher degrees of ionization of the derivatives [16]. Thus, this study proposed to better explore the effect of the degree of ionization of the polycations on the physicochemical and biological properties of the nanocarrier by studying a higher variation of DIPEA grafts (5–55%) on a highly deacetylated chitosan, as shown in Figure 1. The derivatives were prepared using a highly deacetylated and low molecular weight (100 kDa) chitosan sample and further grafted with low densities of PEG (2 kDa) chains to improve the colloidal and biological performance of nanovectors at physiological conditions, i.e., pH 7.4 and ionic strength of 150 mmol L^−1^. The nanocarriers were prepared by the coacervation process and their physicochemical characteristics were evaluated and discussed, focusing on the attributes favorable to transfection, such as small size, positive surface charge, and high biological stability. Finally, the siRNA nanocarriers were subjected to in vitro studies to evaluate their cytotoxicity, cell internalization, and transfection efficiency by the knockdown of GFP and the tumor necrosis factor alpha (TNFα) in HeLa cells and RAW 264.7 macrophages, respectively.

## 2. Results and Discussion

### 2.1. Physicochemical Characterization of Polymers

#### 2.1.1. Polymer Composition

**Figure 2:**
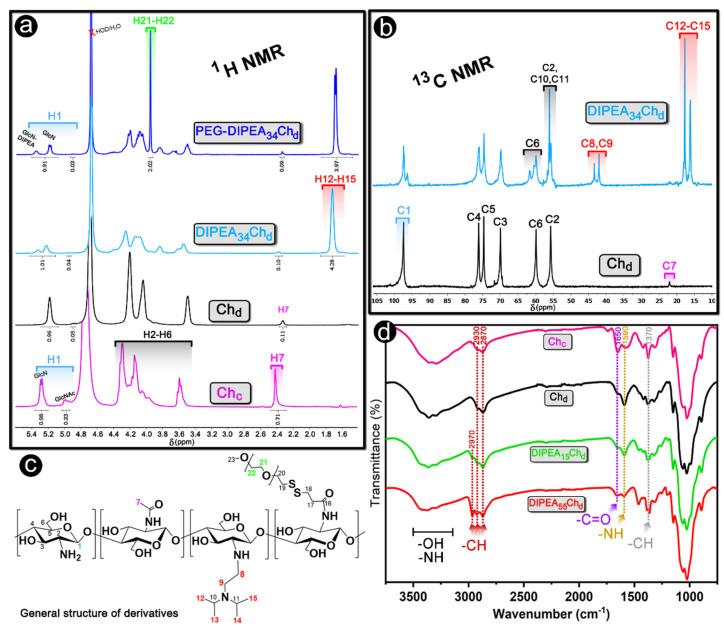
(**a**) Representative ^1^H NMR and (**b**) ^13^C NMR spectra of chitosan and its DIPEA/PEG derivatives associated with (**c**) the general chemical structure of polymers and (**d**) FTIR characterization of chitosan and derivatives.

The main objective of this study was to combine the diisopropylaminoethyl (DIPEA) groups with chitosan to obtain derivatives with increasing degrees of ionization and stable siRNA-nanoparticles at physiological conditions. The advantage of this approach relies on the sensitivity of DIPEA units to pH [21]. Moreover, the grafting onto the primary amino groups of chitosan gives origin to secondary amino groups and this may allow control of the physicochemical properties of the nanoparticles at neutral pH (Figure 1). Representative ^1^H and ^13^C NMR spectra of derivatives are shown in Figure 2 and more detailed spectra are presented in the Supplementary Information (Figures S1–S12).

The degree of deacetylation (*DDA*) was determined using the ^1^H NMR spectra and the Equation (1),

(1) $$DDA=\left(1-\frac{I_{H_{Ac}}}{3I_{H1}}\right)\times 100$$

where *I_HAc_* is the area of the signal of the three methyl hydrogens (H7) of acetylglucosamine units (*δ* 2.4 ppm) and *I_H_*_1_ is the area of the H1 hydrogen signals (*δ* 4.9–5.5 ppm). After deacetylation, the DDA increased from 76% (Ch_c_) to 96% (Ch_d_), which confirms the success of the deacetylation reaction (Table 1). Additionally, the ^13^C NMR spectrum of Ch_c_ has a very low-intensity peak for methyl carbon (C7) of the acetyl group (*δ* 22 ppm), which indicates a high DDA [15,22].

The spectra for derivatives are characterized by the appearance of a strong signal centered at *δ* 1.7 ppm corresponding to methyl hydrogens of the DIPEA groups (Figure 2a). The degrees of substitution by DIPEA (DS_DIPEA_) were determined using Equation (2):

(2) $$DS_{DIPEA}=\frac{I_{H_{DIPEA}}}{12I_{H1}}\times 100\;$$

In this equation, *I_HDIPEA_* is the integral of the signal attributed to the 12 methyl hydrogens of DIPEA (H12-H15) at *δ* 1.6–1.9 ppm. As seen in Table 1, by adjusting the ratio DIPEA-Cl/Ch_d_ in the feed, DIPEA-chitosans with *DS_DIPEA_* in the range of 5 to 55% (mol/mol) could be obtained, which allows a more in-depth study of the effect of DIPEA content on the properties of chitosan as a siRNA nanocarrier. The grafting with DIPEA was also confirmed by the low-field displacement of the H1 signal from *δ* 5.2 to 5.3 ppm and by the emergence of new peaks at *δ* 3.5–4.5 ppm attributed to methine and methylene DIPEA hydrogens (Figure 2a).

The derivatives were also characterized by ^13^C NMR and the appearance of new signals on spectra of the derivatives are compared to the Ch_d_ spectrum in Figure 2b. The new peaks appeared at *δ* 16–18 ppm, *δ* 42–44 ppm, and *δ* 55–57 ppm and are attributed to the methylic (C12–C15), methylene (C8, C9), and methine (C10, C11) carbons from DIPEA, respectively. Additionally, it is worth mentioning that no additional signal was observed at *δ* 65 ppm [23], even for the more substituted derivative (DIPEA_55_Ch_d_), confirming the grafting at the amino groups and a good control of the polymer composition.

Selected DIPEA-derivatives were also grafted with PEG to provide stealth properties to nanoparticles, aiming to avoid the biological interactions responsible for nanoparticles disassembling outside cells. The degrees of substitution by PEG (DS_PEG_) were determined using Equation (3), where the *I_HPEG_* refers to the area under the peak attributed to the 172 methylene protons of PEG repeating units (-OCH_2_CH_2_-), at *δ* 3.9–4.0 ppm [15]. The PEG grafting was also confirmed by the appearance of a small peak at *δ* 3.65 ppm (Figures S7 and S8) attributed to terminal methyl hydrogens (-OCH_3_) of the PEG chain [19]. As shown in Table 1, DS_PEG_ of both grafted derivatives was around 1.5% (mol/mol), and no DIPEA release was observed, which confirms the success of the synthetic route adopted in this study.

(3) $$DS_{PEG}=\frac{I_{H_{PEG}}}{172I_{H1}}\times 100$$

FTIR spectroscopy was also employed to characterize the derivatives and the spectra for chitosans (Ch_d_ and Ch_c_) and some selected derivatives are shown in Figure 2d. When compared to commercial chitosan (Ch_c_), the deacetylated sample (Ch_d_) displays a weak band assigned to the –C=O stretching (1650 cm^−1^) and the –CH symmetrical deformation (1370 cm^−1^). The decreased intensities of both bands are expected, taking into account the deacetylation reaction and this corroborates the NMR results. The substitution by DIPEA was also confirmed by FTIR, i.e., when compared to chitosan (Ch_d_), the FTIR spectra of derivatives displayed a new band at 2970 cm^−1^ assigned to the –CH stretching originating from the DIPEA group (Figure S17). Moreover, the DIPEA grafting led to the decreased intensity for the –NH bending vibration band (1590 cm^−1^) due to the substitution of primary amine groups [24,25].

The average molecular weights (Mw) from chitosan and its derivatives were estimated by GPC (Figure S14) and the results are shown in Table 1. As previously reported [26], the deacetylation reaction led to the depolymerization of chitosan chains due to reaction conditions (high temperature and strong basic solution), which have reduced the Mw from 208 (Ch_c_) to 141 kDa (Ch_d_) and increased the molar-mass dispersity (Đ_M_). Similarly, the molecular weights for derivatives tended to decrease upon DIPEA grafting (especially for the more substituted ones), a sign that main chain degradation took place during the chemical modification of Ch_d_, as earlier reported for DEAE-chitosan [27]. Another relevant effect of MW may be observed in the synthesis of derivatives when the data in Table 1 are compared to previous results reported by our research group [16] for DIPEA-chitosans of higher MW (200 kDa), i.e., under the same experimental conditions, chitosans of high MW need to be fed with more DIPEA-Cl (about 20–30%) to originate DIPEA-chitosans with the same DS_DIPEA_ of derivatives listed in Table 1, which have about 100 kDa.

#### 2.1.2. Compositional Effects on the Degree of Ionization (α) and Buffering Capacity (BC)

**Figure 3:**
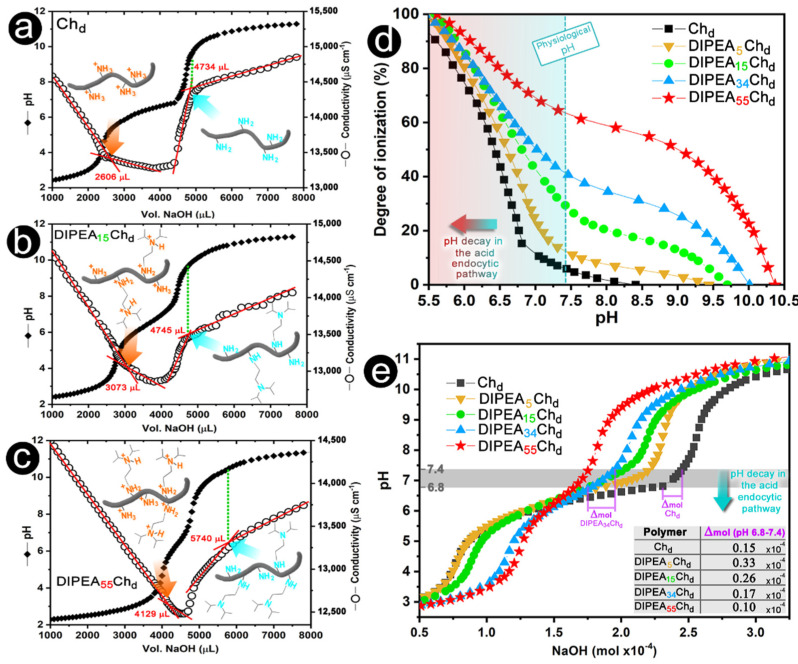
(**a**–**c**) Potentiometric and conductimetric titration of chitosan and derivatives. (**d**) Degree of ionization and (**e**) Buffer capacity of polymers as a function of pH.

The degree of ionization (*α*) of polycations is closely related to the surface charge of their nanoparticles, impacting directly their physicochemical and biological properties. For instance, while a high positive charge density on polycations favors the complexation of nucleic acid, it may increase the toxicity, while nanoparticles with low charge density may have their cell uptake and colloidal stability reduced [28]. In this study, the *α* of derivatives is highly dependent on DIPEA content, especially for higher pH values. The degrees of ionization were determined based on the conductivity titrations of the derivatives. The conductivity traces (Figure 3a–c) allow the detection of the starting and the ending points for the deprotonation of the amino groups. The last break in the conductivity traces corresponds to the complete deprotonation of the tertiary amino groups, which occurs near a pH of 10 and this is consistent with a pKa of 8.5 reported for diisopropylamino groups [29]. The degrees of ionization calculated using Equation (4) (Section 3.2.8) are shown in Figure 3d and reveal that, at physiological pH (7.4), the low *α* of chitosan (6.1%) increased to 13%, 29%, 41%, and 64% for DIPEA_5_Ch_d_, DIPEA_15_Ch_d_, DIPEA_34_Ch_d_, and DIPEA_55_Ch_d_, respectively. In addition, it is worth mentioning that at pH 6.4 deacetylated chitosan is about 50% ionized, in accordance with the reported pKa value for its primary amine groups [5]. Due to the grafting by DIPEA groups, derivatives had a clear increase in their solubility at neutral pH when compared to unsubstituted chitosan that formed visible aggregates (Figure S16 Supplementary Information). It has been reported that the interaction of nanoparticles with the endosomal membrane plays an important role in the endosomal escape rate [30,31]. Thus, decreases in pH during the endocytosis [32] may increase the surface charge of nanoparticles, favoring their interaction with the endosomal membrane and the release process.

Considering that chitosan nanoparticles are internalized mainly via acid endocytic pathways [19,33] and that polycations that having great buffering capacities (BC) may provide an enhanced endosomal escape of the therapeutic genes by the proton sponge hypothesis [34], the BC of DIPEA-chitosan derivatives was also qualitatively evaluated (Figure 3e). Except for DIPEA_55_Ch_d_, all derivatives displayed a BC higher or similar to unmodified chitosan in the pH range from 7.4 to 6.8 (highlighted in Figure 3e), which corresponds to an initial pH decay during the acidic endocytic pathway [35]. The improvement of BC in this pH range may be promising in providing siRNA with an earlier endosomal escape.

### 2.2. Effect of the Degree of Ionization on the Physicochemical Properties of Nanoparticles

#### 2.2.1. Electrophoresis

The polycation–siRNA interaction and the coacervation process to form nanoparticles is mainly driven by electrostatic attraction between the negatively charged siRNA molecules and the polycations [36]. The siRNA loading is very dependent of charge ratios (N/P ratio) in the nanoparticle core, i.e., if there are not enough positively charged amines to neutralize the phosphate groups of the nucleic acid, not all of the siRNA will be efficiently loaded by the vector and the resulting complex will have a negative charge [5,37]. Hence, an electrophoretic mobility study can be used to detect compositions (charge ratios) at which the siRNA is not efficiently complexed, which would result in formulations with low therapeutic potential [38].

**Figure 4:**
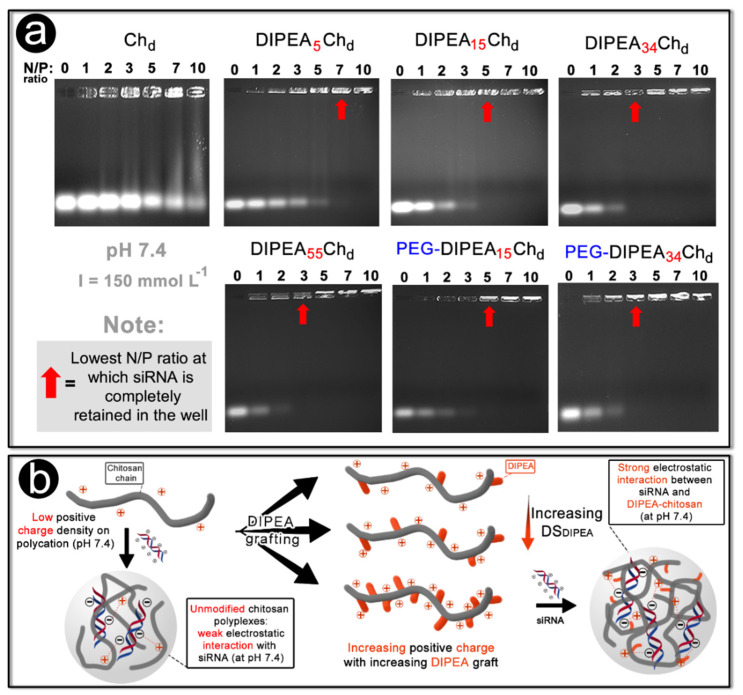
(**a**) Gel electrophoresis of polymer/siRNA nanoparticles at an increasing N/P ratio in phosphate buffer (pH 7.4 and I = 150 mmol L^−1^). The red arrows indicate the complete complexation of siRNA. (**b**) Schematic representation of the effect of DIPEA grafting on chitosan to formulate the siRNA nanocarriers.

At pH 7.4, the deacetylated chitosan (Ch_d_) was not able to retain the siRNA in any of the N/P ratios studied and siRNA was released from all wells (Figure 4a). However, for nanoparticles formulated with DIPEA_5_Ch_d_, DIPEA_15_Ch_d_, DIPEA_34_Ch_d_, and DIPEA_55_Ch_d_, siRNA was completely retained at decreasing N/P ratios (7.0; 5.0; 3.0, and 3.0, respectively) which is consistent with the increasing degrees of ionization for these polycations (Figure 4b). The similar siRNA release for nanoparticles formulated with DIPEA_34_Ch_d_ and DIPEA_55_Ch_d_, even having different ionization at pH 7.4, suggests that besides electrostatic interaction, conformational changes on the polymer chain may affect the condensation process and the strength of interaction.

The PEGylated derivatives (PEG-DIPEA_15_Ch_d_ and PEG-DIPEA_34_Ch_d_) had the same siRNA release behavior as non-PEGylated ones (Figure 4a), indicating that DIPEA substitution enabled chitosan to dampen the negative effect associated with PEGylation, i.e., in general there is a decrease in the strength of the interaction between polycations and siRNA due to the steric hindrance exerted by the PEG chains [5,20].

Overall, these results make clear the positive effects of DIPEA grafting showing the gradual strengthening of the siRNA/polycation interactions, which may be an advantage compared to chitosan that, under physiological pH, interacts weakly with siRNA molecules providing unstable particles at low N/P ratios.

#### 2.2.2. Effect of the Degree of Ionization and PEGylation on Nanoparticle Properties: Size, Morphology, Surface Charge, and Colloidal Stability of Nanocarriers

The control of the surface charge, size, and stability of nanocarriers is pivotal for obtaining promising vectors for gene therapy. In general, siRNA nanocarriers with sizes smaller than 500 nm, slightly positive zeta potentials, and the ability to avoid unwanted interactions during the in vitro application are more prone to efficiently transport the siRNA, to be internalized by cells and to provide high knockdown levels [39,40]. For chitosan nanoparticles, these characteristics depend greatly on the N/P ratio, pH, and ionic strength in which they are prepared [18,41].

**Figure 5:**
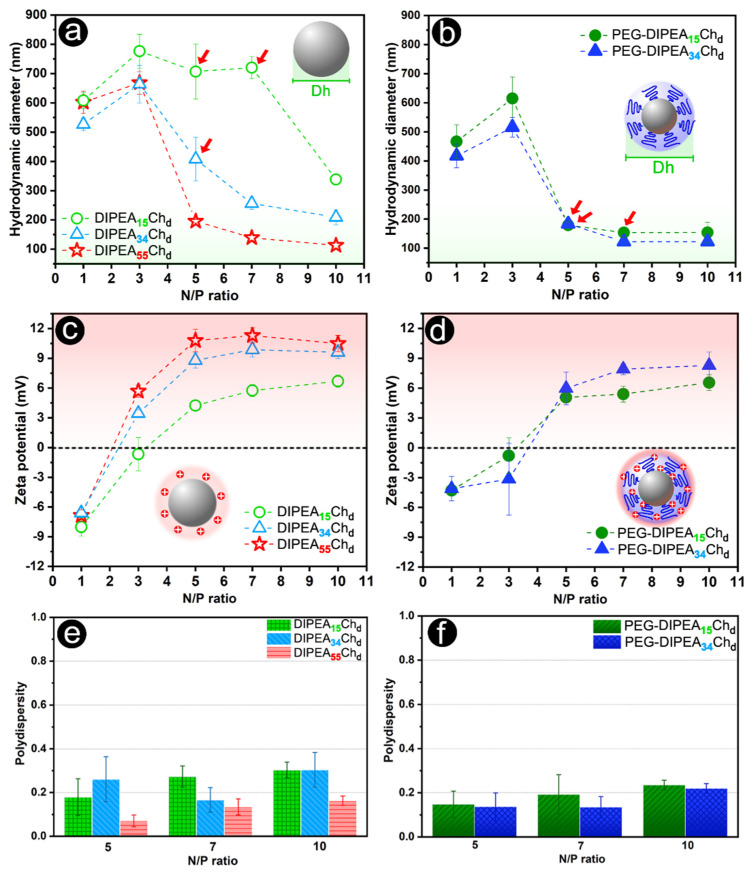
(**a**,**b**) Hydrodynamic diameter, (**c**,**d**) Zeta potential, and (**e**,**f**) polydispersity of nanoparticles assembled with DIPEA (**left**) and DIPEA/PEG (**right**) derivatives with siRNA at an increasing N/P ratio in phosphate buffer (pH 7.4 and I = 150 mmol L^−1^). The red arrows (**a**,**b**) indicate some changes in the size of PEG/DIPEA-nanoparticles compared to non-PEGylated vectors.

To adjust the properties of the nanoparticles for in vivo applications, their formulations were prepared and evaluated at pH 7.4 and ionic strength of 150 mmol L^−1^. For the siRNA carriers based on DIPEA-chitosan, the N/P ratios needed to obtain vectors on a nanoscale decreased with increasing DS_DIPEA_. Concurrently, the zeta potential (ζ) of nanocarriers increases with the DIPEA content, in close agreement with the degree of ionization of the polycations (Figure 5). Nanoparticles smaller than 250 nm were obtained only when the N/P ratio was positive, i.e., the condensation process is efficient only after negative charges of siRNA molecules had been completely neutralized. Moreover, the PEG graft induced changes to ζ and mainly to the hydrodynamic diameter (Dh) of nanoparticles and, also, nanoparticles formulated with PEGylated derivatives were smaller than 200 nm, using 50% less polymer than the non-PEGylated ones (Figure 5a vs. Figure 5b). These nanoparticles had low polydispersities (Figure 5f), and values smaller than 0.2 were often obtained. Overall, while nanoparticles formulated with PEGylated-only chitosan (PEG-Ch_d_) formed complexes composed of large aggregates with a negative charge surface (Figure S18), the derivatives have formed nanoparticles with promising sizes (100–200 nm) and zeta potentials (6–12 mV) to be applied as vectors for gene therapy. Notably, the importance of the degree of ionization (*α*) of these derivatives for obtaining nanovectors of adequate Dh and ζ was also observed in a previous study [16], in which it was shown that high MW DIPEA-chitosans with low DS_DIPEA_ (15%) are more prone to formulate efficient siRNA nanocarriers (with small size, positive ζ, and improved stability) at pH 6.3 (*α* ≥ 80%) than at pH 7.4 (*α* < 35%), due to an increasing degree of ionization with decreasing pH.

**Figure 6:**
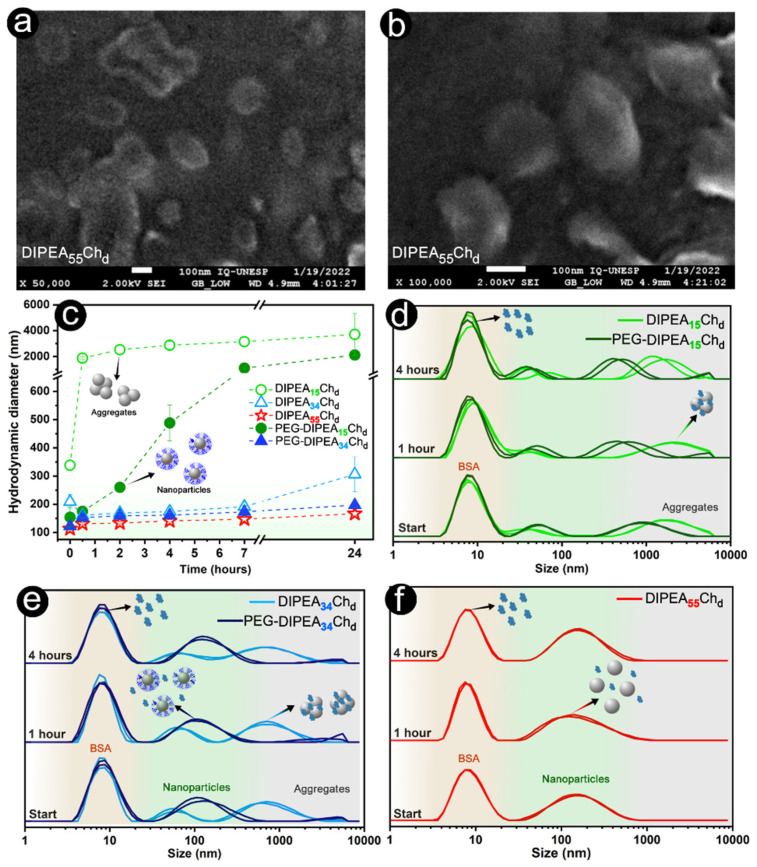
(**a**,**b**) Representative SEM-FEG images of DIPEA_55_Ch_d_/siRNA nanoparticles. (**c**) Hydrodynamic diameter of nanocarriers as a function of time. (**d**–**f**) Representative distributions of hydrodynamic diameter of nanoparticles in the presence of BSA (40 g L^−1^) over time. All nanoparticles were prepared at N/P ratio 10 under physiological conditions of pH (7.4) and ionic strength (150 mmol L^−1^).

SEM-FEG microscopy images (Figure 6a,b) pointed to nanoparticles with spherical-like morphologies and sizes in accordance with the light scattering analysis (100–200 nm), results that are very close to those reported for siRNA nanoparticles formulated with DEAE-chitosans at lower pH values [15,17]. Spherical nanoparticles may have favored cellular uptake in comparison to cubical and rod-shaped nanoparticles [42]. Additionally, the cellular uptake may be enhanced by using positively charged nanoparticles that interact with the endosomal membrane during their internalization [43]. When the DIPEA_34_Ch_d_ nanoparticles formulated at N/P ratio 10 were evaluated at pH 6.3 (early endosome pH), their zeta potential (17.6 mV) was almost twice as high as that at pH 7.4 (9.6 mV), which suggests a favored interaction with the internal endosomal membrane during the acid internalization pathway, in agreement with the ionization results discussed above (Figure S19).

To evaluate the stability of nanoparticles in solution, their hydrodynamic diameter was monitored over time in the presence and absence of bovine serum albumin (BSA). At N/P ratio 10, nanoparticles displayed slightly positive zeta potentials (Figure 5c,d), Dh smaller than 250 nm (Figure 5a,b) and a complete siRNA entrapment (Figure 4a). So, this N/P ratio was chosen for the stability studies carried out under physiological conditions (pH 7.4 and ionic strength of 150 mmol L^−1^), as shown in Figure 6. Contrary to deacetylated chitosan, which forms large aggregates at pH 7.4 [15], the DIPEA-derivatives formed siRNA nanocarriers with good colloidal stability proportional to the DS_DIPEA_ and further improved by the PEGylation. In the absence of BSA, DIPEA_15_Ch_d_ nanoparticles displayed poor stability characterized by the occurrence of large aggregates (greater than 1000 nm) in the first 30 min after their preparation. Although the PEGylation version (PEG-DIPEA_15_Ch_d_) had prolonged the stability of nanoparticle sizes for up to 2 h of analysis, after 24 h, the sizes increased to micrometers (Figure 6c). On the other hand, the most substituted chitosans (PEG-DIPEA_34_Ch_d_ and DIPEA_55_Ch_d_) were able to keep their small sizes (100–200 nm) even after 24 h of analysis, with polydispersities lower than 0.4, suggesting that degrees of ionization higher than 40% are more appropriate to confer stability at neutral pH at physiological ionic strength (Figure S20).

The adsorption of proteins is one of the major obstacles faced by nanoparticles during their biodistribution, since it may alter their physicochemical properties and favor their destruction via an opsonization process that lowers the vector transfection efficacy [44]. Hence, the interaction between nanoparticles and albumin (40 g L^−1^) was probed by monitoring the size distribution of nanoparticles/BSA solution over time under physiological conditions [45]. The experiment was performed using the most substituted DIPEA-chitosan derivatives and their PEGylated versions. The nanoparticle size traces show a BSA peak centered at 8 nm and the non-PEGylated version of DIPEA_34_Ch_d_ nanoparticles showed a peak centered at 600 nm corresponding to BSA/nanocarrier aggregates (Figure 6d–f), while, for the PEG-DIPEA_34_Ch_d_, no aggregates were observed and the sizes remained between 100 and 200 nm, even after four hours of study, indicating an inhibition of the BSA/nanoparticle interactions (Figure 6e). The BSA adsorption is expected considering that the number of negatively charged groups on the BSA molecule increases with pH, therefore interaction with positive surfaces cannot be neglected, since the BSA charge at pH 7.4 is slightly negative [46]. Interestingly, the size distribution of nanoparticles formulated by the most substituted derivative (DIPEA_55_Ch_d_) was minimally affected by BSA presence, i.e., the peak attributed to nanoparticles was kept centered at 100–200 nm and no larger structures were observed, even after four hours of incubation (Figure 6f). These results indicate that DIPEA helps to maintain the structure of the nanoparticles and the vectors with the highest DIPEA content are the most promising to remain stable in biological systems.

### 2.3. In Vitro Studies of Polymers and Nanoparticles

#### Cytotoxicity, Cellular Uptake and Gene Knockdown

**Figure 7:**
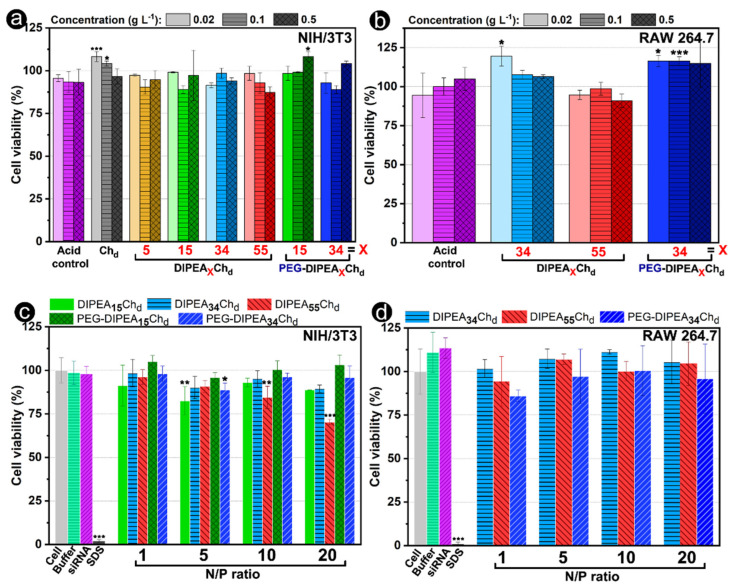
Viability of (**left**) NIH/3T3 and (**right**) RAW 264.7 cells in (**a**,**b**) the presence of increasing polymer concentration and (**c**,**d**) in the presence of nanocarriers formulated at variated N/P ratios. Cell viability was evaluated by an MTS assay and expressed by comparison with the non-treated cells (100% of viability). Statistical analysis was performed using the unpaired Student test (at a significance level of 0.05) and comparisons are shown between (**a**,**b**) the same concentrations of acid controls and polymers and between (**c**,**d**) the non-treated cells (cell) and the cells treated by nanoparticles/other controls. * *p* < 0.05, ** *p* < 0.01, *** *p* < 0.001, no symbol = no significant differences between the means.

The cytotoxicity is one of the essential biological properties that limits the application of polycations as nucleic acid carriers. While non-viral vectors based on synthetic polycations such as PEI, poly(amidoamine) and poly(B-amino ester) may exhibit a more prominent cytotoxicity [47,48,49], chitosan is known for its good cytocompatibility [5]. The cytotoxicity of chitosan derivatives and their nanoparticles were evaluated in RAW 264.7 and NIH/3T3 cell lines using the MTS assay (Figure 7). Both cell lines displayed viabilities close to 100% even after their treatment with polycation concentrations up to 0.5 g L^−1^. For nanoparticles, the viabilities remained above 80% after their incubation with nanoparticles formulated at an N/P ratio of 10. Moreover, compared to previous studies, while 0.3 g L^−1^ of chitosan substituted by 57% of DEAE led to cell viabilities at about 60%, in the presence of a DIPEA_55_Ch_d_ (0.5 g L^−1^) derivative, they remained near to 90%, which suggests that DIPEA derivatives are less cytotoxic and their vectors more suitable for gene therapy [50].

**Figure 8:**
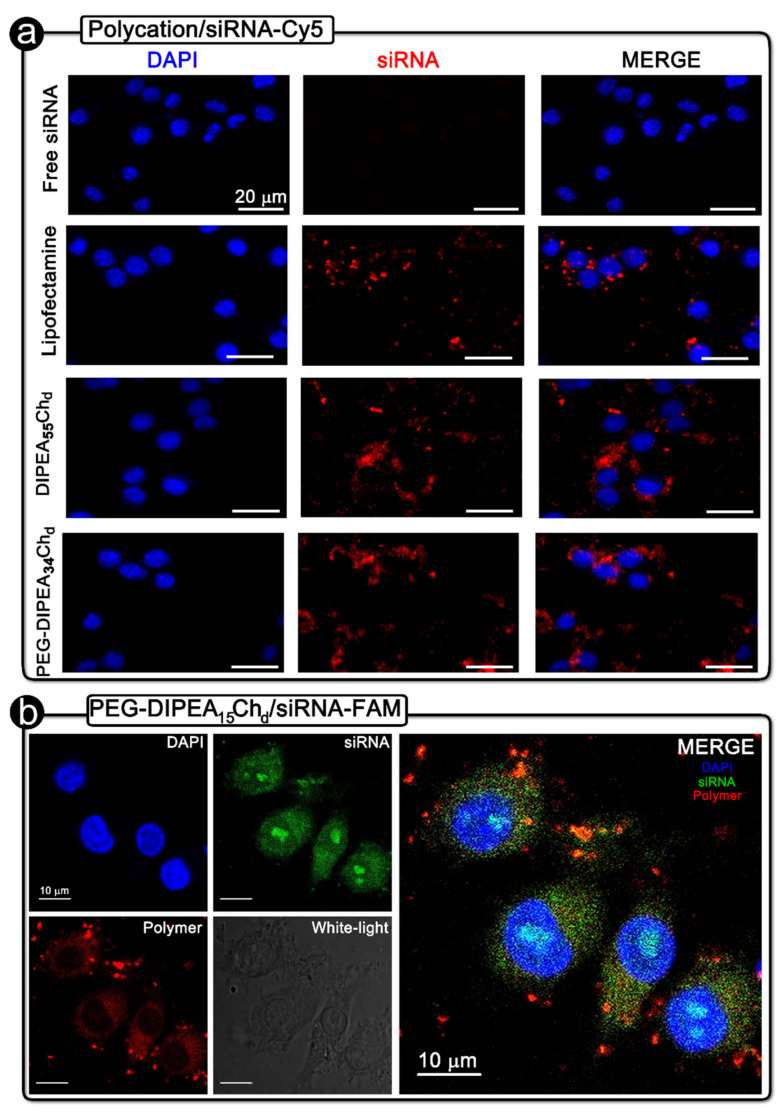
Confocal microscopy images of RAW 264.7 macrophages after their treatment (**a**) with siRNA-Cy5 only or carried by the lipofectamine or by the DIPEA-derivatives, (**b**) and with nanocarriers formulated by siRNA-FAM and RITC/PEG-DIPEA_15_Ch_d_ derivative. All nanocarriers were formulated at N/P ratio 10 under physiological conditions (pH 7.4 and I = 150 mmol L^−1^).

Aiming at evaluating the internalization of the nanocarriers by RAW 264.7 macrophages, the vectors were loaded with siRNAs labeled with fluorophores groups (FAM or Cy5) and the cells were evaluated by confocal microscopy (Figure 8). Due to its size and negatively charged backbone, the free siRNA was not able to enter the cells (Figure 8a upper panel). However, when carried by lipofectamine and by chitosan derivatives, red dots (siRNA-Cy5) could be clearly observed around the nuclei of cells. Additionally, unlike lipofectamine, the siRNA carried by DIPEA-chitosans tended to be distributed more throughout the cytoplasm of the cell. The same behavior was observed for siRNA-FAM transported by PEG-DIPEA_15_Ch_d_ labeled with RITC (Figure 8b), i.e., the siRNA (green dots) extended throughout the whole cytoplasm area, as well as the polycation labeled with rhodamine (red dots). Moreover, the merged image revealed some yellow dots in the cytoplasm, provided by the green (siRNA) and red (polymer) dots overlap, which indicates the presence of intact nanoparticles in addition to the presence of free siRNA and polymer. This result suggests that the nanoparticles can be disassembled at different times inside the cell, so the siRNA is being continuously released by the nanoplatforms developed in this study.

**Figure 9:**
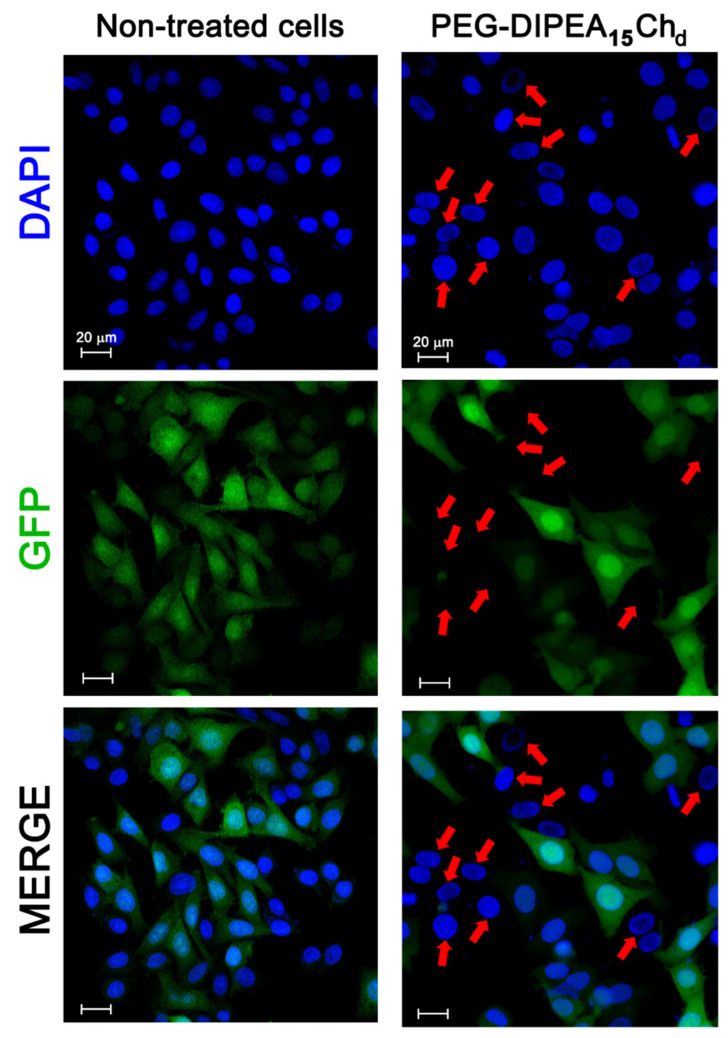
Confocal microscopy images of HeLa-GFP cells stained by DAPI after no vector treatment (non-treated cells) and after their treatment with PEG-DIPEA_15_Ch_d_ nanoparticles loaded with siRNA anti-GFP at N/P ratio 10 (pH 7.4 and I = 150 mmol L^−1^). The red arrows indicate cells at which there was no GFP observed. Scale bar: 20 μm.

**Figure 10:**
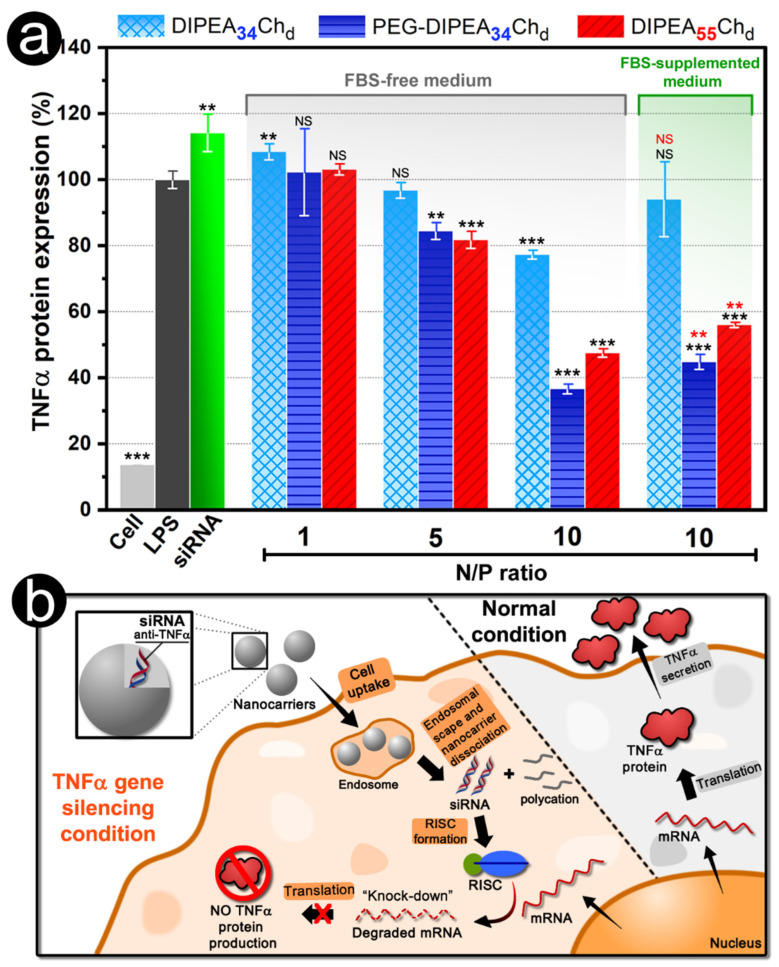
(**a**) Transfection study based on the expression of TNFα protein (measured via ELISA assay) by RAW 264.7 cells after their treatment with nanoparticles loaded with siRNA anti-TNFα (*n* = 3). The percentage of TNFα protein production was expressed by comparison with the cells only treated with LPS (100% TNFα protein production). (**b**) Schematic representation of nanoparticle-mediated TNFα knockdown. Note that all nanocarriers were formulated under physiological conditions of pH (7.4) and ionic strength (150 mmol L^−1^). Statistical analysis (**a**) was performed using the unpaired Student test (at a significance level of 0.05) and comparisons between the only LPS-treated cells and the cells treated by siRNA/nanoparticles (black symbols) are shown and, also, between the same samples in the presence and absence of FBS (red symbols). ** *p* < 0.01, *** *p* < 0.001, and NS = no significant differences between the means.

The potential of DIPEA-chitosan derivatives as siRNA nanocarriers was evaluated by the GFP and TNFα knockdown in HeLa and RAW 264.7 cells, respectively (Figure 9 and Figure 10). After the treatment of HeLa-GFP with PEG-DIPEA_15_Ch_d_ nanoparticles loaded with siRNA anti-GFP, GFP was not visible in several cells, as indicated by the red arrows in the confocal microscopy images (Figure 9). Moreover, when the GFP area in the images were measured and normalized by the DAPI area, a reduction of about 30% in GFP expression was seen for HeLa treated with PEG-DIPEA_15_Ch_d_ nanoparticles in comparison with non-treated cells, a value very close to the lipofectamine result (Figure S23). This result strongly suggests the efficient internalization of carriers displayed in Figure 8b, and reinforces the potential of PEG-DIPEA_15_Ch_d_ as a siRNA nanocarrier.

The nanoparticles formulated with the most substituted derivatives (DIPEA_34_Ch_d_ and DIPEA_55_Ch_d_) and that have exhibited the most promising physicochemical properties, i.e., improved siRNA condensation, small sizes, positive surface, and enhanced colloidal stability, were tested for their ability to knockdown the TNFα cytokine (Figure 10a). The expression of TNFα cytokine by RAW 264.7 cells was evaluated using the ELISA assay and compared with cells treated with LPS only (100% of TNFα expression). In general, the results showed that gene knockdown is dependent on the N/P ratio, DS_DIPEA_, and the PEG grafting. The transfection efficiency mediated by non-PEGylated derivatives (DIPEA_34_Ch_d_ and DIPEA_55_Ch_d_) was greatly dependent on the N/P ratio and a significant knockdown of TNFα was observed only for N/P ≥ 5.0. These results correlate well with the physicochemical properties observed for nanoparticles, which exhibited positive zeta potentials of about +10 mV and Dh values in the range of 100–250 nm only for N/P ratios higher than 5 (Figure 5). In addition, it is noteworthy that, although the physicochemical properties of the nanoparticles were mainly influenced by the degree of ionization of the polycations (as shown in the sections above), all DIPEA-chitosans showed buffering capacity at endosomal pH range (Figure 3), thus the increase of polymer concentration to formulate the nanocarriers at high N/P ratios may favor the endosomal escape of the nanovectors by the proton sponge hypothesis, since the buffering capacity is proportional to the non- protonated amino groups at neutral pH. At N/P ratio 10, the increase in DS_DIPEA_ content, from 34 to 55%, promoted a decrease of about 30% in TNFα expression. Besides, PEGylation provided a significant improvement in transfection efficiency of DIPEA_34_Ch_d_ and the knockdown was increased from 25% to 65% (N/P ratio 10), suggesting that both the decrease in size and the increase in colloidal stability were responsible for the higher efficiency of the PEG-DIPEA_34_Ch_d_ nanoparticles. Moreover, in the presence of the FBS proteins, PEG-DIPEA_34_Ch_d_ and DIPEA_55_Ch_d_ derivatives built siRNA nanoparticles that were able to reduce TNFα expression by 50–65% when compared to LPS-treated cells only, which is consistent with their resistance to protein adsorption displayed in Figure 6, and with their significant cell uptake observed by confocal microscopy (Figure 8). On balance, these results have confirmed that chitosan can be associated with DIPEA and PEG groups for formulating promising siRNA nanocarriers at physiological conditions (pH 7.4, I = 150 mmol L^−1^) that are worthy of evaluation in in vivo studies.

## 3. Materials and Methods

### 3.1. Materials

The commercial chitosan (Ch_c_) was obtained from Polymar (Fortaleza, Brazil). O-(2-mercaptoethyl)-O-methyl-polyethylene glycol (PEG-SH) 2 kDa, 2-Chloro-N,N-diisopropylethylamine hydrochloride (DIPEA-Cl), bovine serum albumin (BSA), cellulose membrane dialysis of MWCO 14 kDa, deuterium chloride (DCl), deuterium oxide (D_2_O), high glucose DMEM medium, fetal bovine serum (FBS), MISSION^®^ siRNA labeled with cyanine (Cy5) or 5′-carboxyfluorescein (FAM), N-Succinimidyl 3-(2-pyridyldithio) propionate (SPDP), rhodamine B isothiocyanate (RITC), and sodium dodecyl sulfate (SDS) were purchased from Sigma-Aldrich (St Louis, MO, USA). EDTA sodium salt, monobasic and dibasic phosphate salts, potassium chloride, sodium chloride, sodium hydroxide, and tris(hydroxymethyl) aminomethane were purchased from Dinâmica (São Paulo, Brazil). Dimethylsulfoxide (DMSO), glacial acetic acid, hydrochloric acid, and methanol were purchased from Synth (Diadema, Brazil). Antibiotic-antimycotic solution (10,000 units mL^−1^ of penicillin, 10 g L^−1^ of streptomycin, and 25 μg/mL of amphotericin B), dialysis membrane of MWCO 3.5 kDa, Lipofectamine™ 2000, siRNA anti-GFP, and siRNA anti-TNFα sequence (5′-3′) sense CGUCGUAGCAAACCACCAAtt, and antisense UUGGUGGUUUGCUACGACGtg were purchased from Thermo Fisher (Madison, WI, USA). RAW 264.7 [15,51] and HeLa-GFP [52] cell lines were obtained from BCRJ (Banco de Células do Rio de Janeiro, Brazil). NIH/3T3 fibroblasts [16,53] were obtained from ATCC^®^ (Virginia, USA). A CellTiter96^®^ Aqueous One Solution kit composed of 3-(4,5-dimethylthiazol-2-yl)-5-(3-carboxymethoxyphenyl)-2-(4-sulfophenyl)-2H-tetrazolium (MTS) and a phenazine ethosulfate (PES) was purchased from Promega Corporation (Fitchburg, MA, USA). A Murine TNFα standard TMB ELISA development kit was obtained from Peprotech^®^ (Cranbury, NJ, USA).

### 3.2. Polymers: Synthesis and Characterization

#### 3.2.1. Deacetylated Chitosan

The commercial chitosan (Ch_c_) was deacetylated as previously described [54] to obtain the starting chitosan (Ch_d_), which was then used for the synthesis of derivatives. Briefly, 5.0 g of Ch_c_ was solubilized in 250 mL of acetic acid (2%, *v*/*v*) under magnetic stirring overnight. Next, 300 mL of NaOH (50%, *w*/*v*) was added to the solution to obtain a finely divided aqueous suspension of chitosan which was heated (100 °C) and kept in an N_2_ atmosphere. After 90 min under intense magnetic stirring in a reflux system, the reaction mixture was poured into 4 L of preheated water (50 °C) and allowed to cool at room temperature. The resulting deacetylated sample was washed several times with deionized water by the decantation process until neutral pH. After that, the precipitated chitosan was recovered by filtration, solubilized in acetic acid (250 mL; 2%, *v*/*v*), and the deacetylation process was repeated once more aiming to obtain a high DDA for the starting chitosan. After sedimentation and filtration, the deacetylated chitosan (Ch_d_) was dried by lyophilization.

#### 3.2.2. DIPEA-Chitosan Derivatives

The different DIPEA derivatives were obtained by adjusting the ratios of 2-Chloro-N,N-diisopropylethylamine hydrochloride (DIPEA-Cl) to glucosamine of deacetylated chitosan (Ch_d_) according to Table 1. The procedure adopted for DIPEA_34_Ch_d_ synthesis is described below. First, 584 mg of Ch_d_ was solubilized under magnetic stirring in 36 mL of 0.1 mol L^−1^ HCl overnight. Then, the solution was heated to 70 °C and the pH was adjusted to pH 12 with 5 mol L^−1^ NaOH. Next, 526 mg of DIPEA-Cl was added into the reaction mixture and kept under intense magnetic stirring for 90 min (at 70 °C). During the reaction time, the pH was monitored and 5 mol L^−1^ NaOH was added whenever necessary to maintain the pH at 12. The polymer was purified by dialysis (MWCO 3.5 kDa) on the first day against NaOH (0.05 mol L^−1^) and on the following days against deionized water, with a daily change of solvent until neutral pH. Finally, the polycation was recovered by lyophilization. All other DIPEA-chitosan derivatives were obtained by the same procedure.

#### 3.2.3. PEGylated Chitosan Derivatives by Disulfide Bonds

The chitosan derivatives were grafted with 2 kDa O-(2-mercaptoethyl)-O-methyl-polyethylene glycol (PEG-SH) by activation with *N*-succinimidyl 3-(2-pyridyldithio) propionate (SPDP) [15]. The procedure for PEG-DIPEA_34_Ch_d_ synthesis is described below. Initially, 200 mg of DIPEA_34_Ch_d_ were solubilized with 10 mL of acetic acid (0.03 mol L^−1^) and diluted with PBS buffer (pH 7.4) to obtain a polymer concentration of 10 g L^−1^. Next, the pH of the mixture was adjusted to 7.4 with NaOH (5 mol L^−1^) and 3.8 mg of SPDP (solubilized in 1 mL of DMSO) was added dropwise into the polymeric solution and kept under magnetic stirring at room temperature. After 3 h, 24.5 mg of PEG-SH (solubilized in 1 mL of PBS) was added to the reaction mixture and stirred for another 16 h at 40 °C. The derivative was purified by dialysis (MWCO 14 kDa) against PBS and an NaOH solution (pH 8–9) for three days with a daily change of solvent. Finally, the PEG-DIPEA_34_Ch_d_ was recovered by lyophilization. Other PEG-grafted derivatives were obtained by the same procedure. In this reaction, the molar ratio between the PEG-SH and the polymer primary amine groups (NH_2_) was fixed at 0.02 and the PEG-SH/SPDP ratio was fixed at 1.0 (Table 1).

#### 3.2.4. Rhodamine-Labeled Chitosan Derivative

Aiming to evaluate the internalization of the nanoparticles by the cells, a chitosan derivative was labeled with rhodamine B isothiocyanate (RITC) following a previously reported method [55,56]. Then, 120 mg of PEG-DIPEA15Chd was solubilized in 15 mL of acetic acid (0.1%, *v*/*v*) and the pH of the solution was set at 6.2 by the addition of 0.1 mol L^−1^ NaOH. Next, 3.5 mL of a methanolic RITC solution (0.2%, *m*/*v*) was added dropwise to the mixture and kept under magnetic stirring in an N_2_ atmosphere for 18 h. After that, the polymer was purified by dialysis (MWCO 3.5 kDa) against NaOH solution (0.05 mol L^−1^) for one day and against deionized water for 10 days, with a daily change of solvent. Finally, the RITC/PEG-DIPEA_15_Ch_d_ was recovered by lyophilization, washed with acetone by a Soxhlet system, and dried at room temperature. The degree of substitution by RITC (DS_RITC_) on the derivative was determined by UV-visible spectroscopy using the RITC as the standard to build the analytical curve (Figure S13, Supplementary Information), as previously described [56].

#### 3.2.5. Hydrogen (^1^H) and Carbon (^13^C) Nuclear Magnetic Resonance (NMR) Spectroscopy

The polymer compositions were determined by ^1^H NMR spectroscopy using a 400 MHz 400/54 Premium Shielded NMR spectrometer (Agilent Technologies, Santa Clara, CA, USA) at 70 °C, with a 4.0 s acquisition time and relaxation delay of 1.0 s. For each spectrum, about 64 or 128 scans were accumulated. The ^13^C NMR analysis of selected polymers was performed at 20 °C on a 400 MHz Avance II NMR spectrometer (Bruker, Billerica, MA, USA) at a ^13^C-frequency of 150.9 MHz with a 12° pulse width. The acquisition time was 0.4 s with a relaxation delay of 2.0 s. For each ^13^C NMR spectrum, about 20,000 scans were accumulated. In both analyses, the samples were solubilized in DCl/D_2_O (1:20) to obtain a polymer concentration of 10 g L^−1^ (^1^H NMR) or 70 g L^−1^ (^13^C NMR). Before dissolution, the polymers were dried at 60 °C under reduced pressure.

#### 3.2.6. Fourier Transform Infrared (FTIR)

The starting chitosan and selected DIPEA derivatives were analyzed with a Vertex 70 Attenuated total reflectance—Fourier transform spectrometer, (Bruker, Ettlingen, Germany). The polymeric samples were dried at 60 °C under reduced pressure and kept inside a desiccator for four hours before analysis. For each sample, 32 scans were collected at 4 cm^−1^ resolution from 4000 to 400 cm^−1^ and the spectra were analyzed with the OriginPro 8.5 software.

#### 3.2.7. Gel Permeation Chromatography (GPC)

The GPC was used to estimate the molecular weights and molar-mass dispersity (*Đ_M_*) of the polymers, using monodisperse pullulan samples with molecular weights ranging from 6.2 to 805.0 kDa as standard to build the analytical curve (Figure S14, Supplementary Information). The measurements were carried out on an LC-20A chromatograph with RID-10A refractive index detection (Shimadzu, Kyoto, Japan). Two linked columns (SB-803 HQ and SB-805-HQ, Shodex, New York, NY, USA), with dimensions of 0.8 cm × 30 cm, were applied for the separation of the polymeric chains. The polymers were dissolved in acetic buffer (0.3 mol L^−1^ acetic acid/0.2 mol L ^−1^ sodium acetate) pH 4.5 at a final concentration equal to 5 g L^−1^. The analysis was performed using the same acetic buffer as eluent with a flow rate of 0.8 mL min^−1^ and at a temperature of 40 °C.

#### 3.2.8. Ionization Degree and Buffering Capacity

The degree of ionization (*α*) and the buffering capacity (BC) were evaluated by conductometric and potentiometric titrations. First, the proper amount of polymer (previously dried under reduced pressure at 60 °C) needed for the 2.4 × 10^−4^ mol of amine groups were solubilized in 40 mL of HCl (0.1 mol L^−1^) with the ionic strength adjusted to 150 mmol L^−1^ with NaCl. Next, the polymeric solutions were titrated with a standardized NaOH (0.1 mol L^−1^) solution and the pH and conductivity were monitored. The BC was estimated by the NaOH consumed in a specific pH range, based on the acid-base titration curve. The *α* as a function of pH was estimated using Equation (4), where *V_X_* is the volume of NaOH added to achieve a specific pH value and *V_1_* and *V_2_* are the volumes at the beginning (*α* = 100%) and the end (*α* = 0) of the deprotonation process, respectively. Both titrations were performed at the same time to obtain *V_1_* and *V_2_* (conductometric) and pH (potentiometric).

(4) $$\alpha =[1-\frac{\left(V_{X}-V_{1}\right)}{\left(V_{2}-V_{1}\right)}]\times \;100$$

### 3.3. Nanoparticles: Formulation and Characterization

#### 3.3.1. siRNA Nanoparticle Formulation

Nanoparticles were self-assembled following the complexation method [19,57]. First, a stock solution (approx. 1.0 g L^−1^) was prepared by the solubilization of the polymer sample in 0.1 mol L^−1^ HCl followed by its dilution in a 50 mmol L^−1^ phosphate buffer pH 7.4, whose ionic strength (I) was set to 150 mmol L^−1^ with NaCl. Next, the appropriate volume of this polymeric stock solution was vortexed with siRNA in phosphate buffer to build the nanocarriers, which were kept at room temperature for 30 min before measurement. The final concentration of siRNA in the nanocarrier solution varied from 0.37 to 3.70 μmol L^−1^, depending on the analysis. The volume of the polymeric stock solution was adjusted to build nanoparticles at varied *N*/*P* ratios (*N* = amino groups of polymer, *P* = phosphate groups of siRNA), calculated using Equation (5). Where *n_siRNA_* is the number of siRNA moles in the solution and the term 42 refers to the number of phosphate groups per siRNA molecule. The terms *m_pol_* and *mm_pol_* refer respectively to the polymer mass (grams) in the solution and the average molecular mass (g mol^−1^) of repeating units present in the polymer chain. *β* is the mean number of amino groups present in the polymer (for example, each 100 units of DIPEA_34_Ch_d_ have 130 amines, then its *β* is 1.30). The *mm_pol_* and the *β* are found based on polymer composition, which is determined by ^1^H NMR spectroscopy. The *n_siRNA_* was calculated based on siRNA concentration and its molecular mass.

(5) $$\frac{N}{P}=\frac{\left(\frac{m_{pol}}{mm_{pol}}\times \beta \right)}{\;\left(n_{siRNA}\times 42\right)}$$

#### 3.3.2. Electrophoresis Assay: Evaluation of siRNA-Polycation Interaction

The free siRNA and the nanocarriers formulated at an increasing N/P ratio (1 to 10) were applied to 0.8% (*m*/*v*) agarose gel containing ethidium bromide (0.4 μg mL^−1^) for siRNA staining. The volume of nanocarrier solution was fixed at 10 μL and its siRNA concentration was 3.70 μmol L^−1^. Before their application in the agarose gel, the nanocarriers were mixed with 1.6 μL of a loading dye solution (0.25% bromophenol blue and 25% Ficoll^®^ 400, *m*/*v*). The electrophoresis was performed at 80 V for 75 min using TAE as running buffer. Finally, the resulting gel was documented by a UV transilluminator system.

#### 3.3.3. Zeta Potential (ζ) and Hydrodynamic Diameter (Dh) of Nanoparticles

The zeta potential and the hydrodynamic diameter of nanoparticles were measured using a Zetasizer Nano ZS with a red laser (λ = 633 nm) and detection optics at 173° (Malvern Panalytical; Malvern, UK) at 25 °C. The nanocarriers were formulated with varied N/P ratios and the siRNA concentration was fixed at 0.37 μmol L^−1^. The sample volume was set at 1 mL and the results were expressed as the mean ± SD based on three independent replicates. The Dh measurements were based on Z-average size. Aiming to evaluate the colloidal stability of the nanocarriers, they were formulated as described above and their Dh was measured over time at room temperature. Additionally, to evaluate the nanoparticle behavior in the presence of proteins, their size distribution was monitored over time after the addition of bovine serum albumin (BSA) at a final concentration equal to 40 g L^−1^. Between measurements, the samples were kept at 37 °C.

#### 3.3.4. Morphology

The nanoparticles were formulated as described above for the ζ and Dh measurements at an N/P ratio of 10. Next, 1 μL of nanocarrier solution was dripped over a silicon plate that was kept inside a desiccator for solvent evaporation. Then, the nanoparticles were coated with a carbon layer and evaluated by an FSM-6701F Field Emission Gun—Scanning Electron (FEG-SEM) microscope (JEOL, Akishima, Japan). The samples were examined under an accelerating voltage of 2.0 kV.

### 3.4. Biological Assays

#### 3.4.1. Cell Culture

The RAW 264.7 murine macrophages and NIH/3T3 fibroblasts were grown in high glucose DMEM medium supplemented with 10% (*v*/*v*) of fetal bovine serum (FBS) and 1% (*v*/*v*) of the antibiotic-antimycotic solution (RAW 264.7 cells) or penicillin-streptomycin (NIH/3T3 cells). HeLa-GFP cells were grown in high glucose DMEM enriched by FBS (10%), non-essential amino acids (0.1 mmol L^−1^), blasticidin (20 μg mL^−1^), and antibiotic-antimycotic solution (1%). All cell lines were incubated at 37 °C in a humidified chamber containing a 5% CO_2_ atmosphere. The studies were performed with cells at subcultivation ratios from 1:2 to 1:6.

#### 3.4.2. Cytotoxicity of Polymers and Nanoparticles

RAW 264.7 or 3T3/NIH cells were seeded in a 96-well plate at a density of 20,000 cells per well one day before their treatment with the nanoparticles or polymer solutions. Then, for evaluation of polymer cytotoxicity, the culture medium was replaced by 200 μL of polymeric solution diluted in complete medium at increasing concentrations (0.02, 0.1, and 0.5 g L^−1^). For evaluation of nanocarrier cytotoxicity, the cells received 50 μL of nanoparticle solution (formulated in phosphate buffer at varied N/P ratios) plus 150 μL of complete medium. The final concentration of siRNA applied to cells was 200 nmol L^−1^. After 24 h of incubation, the cell viability was evaluated using a CellTiter96^®^ Aqueous One Solution kit (Promega Corporation, Fitchburg, MA, USA) following the manufacturer instructions. For this purpose, the medium was replaced by 100 μL of incomplete medium plus 20 μL of MTS, the cells were incubated for 3 h and then the absorbances were measured at 490 nm on an Elx 808 microplate reader (Agilent Technologies, Winooski, VT, USA). The following controls were used: cells treated with 3% (*m*/*v*) sodium dodecyl sulfate (SDS), DMEM/HCl solution, the phosphate buffer used for nanoparticle formulation, and the non-treated cells. The cell viability was determined by comparison with non-treated cells (100% of cell viability), and the results were expressed as the mean ± SD for three independents assays. Statistical analysis was performed using the unpaired Student *t*-test following the Holm–Sidak method at a significance level of 0.05.

#### 3.4.3. Confocal Microscopy: Cellular Uptake and GFP Knockdown

In the cellular uptake study, RAW 264.7 macrophages were seeded at a density of 6.0 × 10^4^ cells per well in a 24-well plate containing a square glass coverslip at the bottom and kept overnight in incubation. The next day, the cells were washed with PBS buffer prior to the addition of 400 μL of siRNA-nanocarrier solution plus 600 μL of incomplete medium. The nanocarriers were formulated at N/P ratio 10 and the final concentration of siRNA was 50 nmol L^−1^. Here, siRNAs labeled with cyanine 5 (siRNA-Cy5) or 5′-carboxyfluorescein (siRNA-FAM) were used. After 4 h of incubation, the cells were fixed by 1 mL 4% (*m*/*v*) paraformaldehyde for 15 min and nuclei stained by 1 mL of 4′,6-diamidine-2′-phenylindole (DAPI, 1 mg L^−1^) for 10 min. After washing the cells with PBS, the coverslips were mounted on glass slides containing glycerol and the images were captured by an LSM 710 microscope (Zeiss, Oberkochen, Germany) using ZEN 2010 software.

In the GFP knockdown study, HeLa-GFP cells (6.0 × 10^4^ cells/well) were grown in a square glass coverslip as described above for RAW 264.7 macrophages. After washing with PBS buffer, 400 μL of nanoparticles (N/P ratio 10, pH 7.4, and I = 150 mmol L^−1^) plus 600 μL of DMEM were added. In this experiment, the nanoparticles were formulated with siRNA anti-GFP, whose final concentration was 200 nmol L^−1^. After 4 h of incubation, the medium was replaced by complete medium and the HeLa-GFP cells were incubated for another 44 h. Next, the cells were fixed, nuclei stained and analyzed as described for the cellular uptake study of RAW 264.7 macrophages.

#### 3.4.4. Transfection Efficiency Study

The transfection efficiency was evaluated by the knockdown of TNFα protein on RAW 264.7 macrophages treated with nanocarriers loaded with siRNA anti-TNFα, as previously reported [16]. Briefly, RAW 264.7 cells were seeded in a 24-well plate at a density of 1.8 × 10^5^ cells per well and incubated overnight. Then, the cells were washed with PBS buffer and received 450 μL of nanoparticles/siRNA solution plus 1.35 mL of medium (with or without 10% FBS). The nanocarriers were formulated at various N/P ratios (1 to 10) and the final concentration of siRNA was 200 nmol L^−1^. After 5 h of incubation, the medium was replaced by 1 mL of complete medium and the cells were incubated for a further 19 h. After that, the medium was removed and the macrophages received 300 μL of complete medium with lipopolysaccharide (LPS; 100 ng mL^−1^) from Escherichia coli. The plate was incubated for 4 h, then the supernatant was collected, centrifuged (12,000 RCF), and stored (−20 °C) for TNFα quantification using a Murine TNFα Standard TMB ELISA Development kit (Peprotech, Rocky Hill, CT, USA), following the manufacturer instructions. The relative TNFα expression was determined by comparison with cells treated with LPS only (100% of TNFα expression). As controls, cells non-stimulated by LPS and cells treated with siRNA only were used. Experiments were performed in triplicate and expressed as the mean ± SD. Statistical analysis was performed using the unpaired Student *t*-test following the Holm–Sidak method at a significance level of 0.05.

## 4. Conclusions

Diisopropylethylamine-chitosan derivatives with varied compositions were synthesized to develop a potential carrier for siRNA delivery. To overcome the low affinity of chitosan with siRNA at neutral pH, the degree of ionization of the polycations was tuned by the content of diisopropylethylamine groups. Vectors with degrees of ionization varying from 30 to 65% were shown to form positively charged siRNA nanoparticles with good colloidal stability at pH 7.4. The insertion of DIPEA increased the siRNA-polycation interactions and allowed positively charged nanoparticles with small sizes at N/P ratios 5 and 10 to be obtained. Derivatives were also grafted with PEG and stability studies showed that nanoparticles having spherical-like shapes retained their sizes in the range of 100–200 nm with low polydispersities 24 h after their formulation and revealed good stability even in a protein medium. Chitosan-derivatives and their siRNA nanocarriers showed low cytotoxicity (cell viability above 85%) in different cell lines. Confocal microscopy showed a successful uptake of nanocarriers by RAW 264.7 macrophages and a promising ability to silence GFP in HeLa cells. Additionally, the siRNA nanocarriers were able to promote TNFα knockdown in LPS-stimulated macrophages, even in FBS medium, and the results showed close agreement with nanocarrier physicochemical properties. Overall, this study provides new systems based on chitosan with an enhanced siRNA condensation process and highlights the potential of these derivatives for in vivo studies.

## Figures and Tables

**Table 1:** Physicochemical properties of chitosan and its amined derivatives.

Polymer	DIPEA-Cl/NH_2_^i^	PEG-SH/NH_2_ (×10^−2^) ^i^	DDA (%) ^ii^	DS_DIPEA_ (%) ^ii^	DS_PEG_ (%) ^ii^	${\bar{M}}_{w}$ (kDa) ^iii^	*Đ*_M_ ^iii^
Ch_c_			76			208.2	1.92
Ch_d_ ^iv^			96			141.1	3.02
DIPEA_5_Ch_d_	0.14			4.7		113.6	3.26
DIPEA_15_Ch_d_	0.44			15		98.3	2.74
DIPEA_34_Ch_d_	0.76			34		83.0	2.45
DIPEA_55_Ch_d_	0.97			55		90.6	2.71
PEG-DIPEA_15_Ch_d_		2.0		15 ^v^	1.7		
PEG-DIPEA_34_Ch_d_		2.0		34 ^v^	1.3		

^i^ Molar ratios of DIPEA-Cl and PEG-SH relative to the amine groups (NH_2_) from Ch_d_ that were used in the derivatives synthesis. DIPEA-Cl [2-Chloro-N,N-diisopropylethylamine hydrochloride], PEG-SH [O-(2-mercaptoethyl)-O-methyl-polyethylene glycol]. ^ii^ Determined by ^1^H NMR. DDA [Degree of Deacetylation], DS_DIPEA_ [Degree of Substitution by DIPEA] DS_PEG_ [Degree of Substitution by PEG]. ^iii^ Estimated by GPC. ${\bar{M}}_{w}$ [average molecular weight] *Đ*_M_ [polymer molar-mass dispersity]. ^iv^ Starting deacetylated chitosan for DIPEA-synthesis. ^v^ Determined by non-PEGylated derivatives.

## Supplementary Materials

The following supporting information can be downloaded at: https://www.mdpi.com/article/10.3390/md20080476/s1, Figure S1. ^1^H NMR spectrum of Ch_c_ (commercially obtained chitosan). Figure S2. ^1^H NMR spectrum of Ch_d_ (deacetylated chitosan). Figure S3. ^1^H NMR spectrum of DIPEA_5_Ch_d_ derivative. Figure S4. ^1^H NMR spectrum of DIPEA_15_Ch_d_ derivative. Figure S5. ^1^H NMR spectrum of DIPEA_34_Ch_d_ derivative. Figure S6. ^1^H NMR spectrum of DIPEA_55_Ch_d_ derivative. Figure S7. ^1^H NMR spectrum of PEG-DIPEA_15_Ch_d_ derivative. Figure S8. ^1^H NMR spectrum of PEG-DIPEA_34_Ch_d_ derivative. Figure S9. ^13^C NMR spectrum of Ch_d_ (deacetylated chitosan). Figure S10. ^13^C NMR spectrum of DIPEA_15_Ch_d_ derivative. Figure S11. ^13^C NMR spectrum of DIPEA_34_Ch_d_ derivative. Figure S12. ^13^C NMR spectrum of DIPEA_55_Ch_d_ derivative. Figure S13. (a) Analytical curve of Rhodamine B isothiocyanate (RITC) in acetic acid -sodium acetate buffer pH 4.5/methanol (50:50 *v*/*v*) and (b) UV-visible absorbance spectrum of RITC/PEG-DIPEA_15_Ch_d_ used to determinate the DS_RITC_. Figure S14. GPC chromatograms of chitosan and its derivatives. The analytical curve was performed using pullulan standards with molecular weight in the range of 6.2 to 805 kDa. Figure S15. Conductometric and potentiometric titrations of polymeric solutions with NaOH 0.077 mol L^−1^. V1 and V2 refer to the volume of NaOH (mL) to consume the excess of HCl and to deprotonate the amine groups from polymer, respectively. Figure S16. Solubility of Chitosan and its DIPEA derivatives at pH 7.4 and ionic strength of 150 mmol L^−1^. Figure S17. Magnification of selected bands from FTIR spectra of (a) commercial and deacetylated chitosan, and (b,c) DIPEA-derivatives. Figure S18. (a) hydrodynamic diameter, Zeta potential and (b) polydispersity of polyplexes formed with siRNA anti-TNFα PEG-Ch_d_ (pH 7.4, I = 150 mmol L^−1^). Figure S19. (a) hydrodynamic diameter (b) Zeta potential, and (c) size polydispersity of polyplexes formed by DIPEA_34_Ch_d_ (and siRNA anti-TNFα) at different N/P ratios at pH 6.3 (I = 50 mmol L^−1^; Phosphate = 25 mmol L^−1^) and 7.4 (I = 150 mmol L^−1^, Phosphate = 50 mmol L^−1^). Figure S20. Polydispersity of nanoparticles as time function. The nanoparticles were formulated at 10 N/P ratio in a solution with pH 7.4 and I = 150 mmol L^−1^. Figure S21. Size distribution of free albumin (BSA) in phosphate buffer, pH 7.4, and I = 150 mmol L^−1^ (n = 2). Figure S22. Size distribution curves of nanoparticles formulated at N/P ratio 10, pH 7.4, and I = 150 mmol L^−1^ (n = 3). Figure S23. (colorful) Confocal microscopy images of non-treated and treated HeLa-GFP cells with siRNA anti-GFP-nanoparticles of PEG-DIPEA_15_Ch_d_ and Lipofectamine. (black-and-white) Images analyzed by ImageJ software for the white area measurement.

## References

[1] Hu B., Zhong L., Weng Y., Peng L., Huang Y., Zhao Y., Liang X.J. (2020). Therapeutic SiRNA: State of the Art. Signal Transduct. Target. Ther..

[2] de Brito e Cunha D., Frederico A., Azamor T., Melgaço J., da Costa Neves P., Bom A., Tilli T., Missailidis S. (2022). Biotechnological Evolution of SiRNA Molecules: From Bench Tool to the Refined Drug. Pharmaceuticals.

[3] Abu Abed O.S. (2021). Gene Therapy Avenues and COVID-19 Vaccines. Genes Immun..

[4] Zamboulis A., Nanaki S., Michailidou G., Koumentakou I., Lazaridou M., Ainali N.M., Xanthopoulou E., Bikiaris D.N. (2020). Chitosan and Its Derivatives for Ocular Delivery Formulations: Recent Advances and Developments. Polymers.

[5] Cao Y., Tan Y.F., Wong Y.S., Liew M.W.J., Venkatraman S. (2019). Recent Advances in Chitosan-Based Carriers for Gene Delivery. Mar. Drugs.

[6] Iranpur Mobarakeh V., Modarressi M.H., Rahimi P., Bolhassani A., Arefian E., Atyabi F., Vahabpour R. (2019). Optimization of Chitosan Nanoparticles as an Anti-HIV SiRNA Delivery Vehicle. Int. J. Biol. Macromol..

[7] Liang Y., Wang Y., Wang L., Liang Z., Li D., Xu X., Chen Y., Yang X., Zhang H., Niu H. (2021). Self-Crosslinkable Chitosan-Hyaluronic Acid Dialdehyde Nanoparticles for CD44-Targeted SiRNA Delivery to Treat Bladder Cancer. Bioact. Mater..

[8] Sava V., Fihurka O., Khvorova A., Sanchez-Ramos J. (2020). Enriched Chitosan Nanoparticles Loaded with SiRNA are Effective in Lowering Huntington’s Disease Gene Expression Following Intranasal Administration. Nanomed. Nanotechnol. Biol. Med..

[9] Koli U., Nilgiriwala K., Sriraman K., Jain R., Dandekar P. (2019). Targeting Tuberculosis Infection in Macrophages Using Chitosan Oligosaccharide Nanoplexes. J. Nanoparticle Res..

[10] Moraru C., Mincea M., Menghiu G., Ostafe V. (2020). Understanding the Factors Influencing Chitosan-Based Nanoparticles-Protein Corona Interaction and Drug Delivery Applications. Molecules.

[11] Pathomthongtaweechai N., Muanprasat C. (2021). Potential Applications of Chitosan-Based Nanomaterials to Surpass the Gastrointestinal Physiological Obstacles and Enhance the Intestinal Drug Absorption. Pharmaceutics.

[12] Zhang C.G., Zhu W.J., Liu Y., Yuan Z.Q., Yang S.D., Chen W.L., Li J.Z., Zhou X.F., Liu C., Zhang X.N. (2016). Novel Polymer Micelle Mediated Co-Delivery of Doxorubicin and P-Glycoprotein SiRNA for Reversal of Multidrug Resistance and Synergistic Tumor Therapy. Sci. Rep..

[13] Deng Y., Chen J., Zhao Y., Yan X., Zhang L., Choy K., Hu J., Sant H.J., Gale B.K., Tang T. (2016). Transdermal Delivery of SiRNA through Microneedle Array. Sci. Rep..

[14] Nam J.P., Nah J.W. (2016). Target Gene Delivery from Targeting Ligand Conjugated Chitosan-PEI Copolymer for Cancer Therapy. Carbohydr. Polym..

[15] Martins G.O., Segalla Petrônio M., Furuyama Lima A.M., Martinez Junior A.M., de Oliveira Tiera V.A., de Freitas Calmon M., Leite Vilamaior P.S., Han S.W., Tiera M.J. (2019). Amphipathic Chitosans Improve the Physicochemical Properties of SiRNA-Chitosan Nanoparticles at Physiological Conditions. Carbohydr. Polym..

[16] Martinez Junior A.M., de Souza R.H.F.V., Petrônio M.S., Martins G.O., Fernandes J.C., Benderdour M., Tiera V.A.d.O., Tiera M.J. (2022). Double-Grafted Chitosans as SiRNA Nanocarriers: Effects of Diisopropylethylamine Substitution and Labile-PEG Coating. J. Nanostructure Chem..

[17] de Souza R.H.F.V., Picola I.P.D., Shi Q., Petrônio M.S., Benderdour M., Fernandes J.C., Lima A.M.F., Martins G.O., Martinez Junior A.M., de Oliveira Tiera V.A. (2018). Diethylaminoethyl-Chitosan as an Efficient Carrier for SiRNA Delivery: Improving the Condensation Process and the Nanoparticles Properties. Int. J. Biol. Macromol..

[18] Alameh M., Lavertu M., Tran-Khanh N., Chang C.Y., Lesage F., Bail M., Darras V., Chevrier A., Buschmann M.D. (2018). SiRNA Delivery with Chitosan: Influence of Chitosan Molecular Weight, Degree of Deacetylation, and Amine to Phosphate Ratio on In Vitro Silencing Efficiency, Hemocompatibility, Biodistribution, and In Vivo Efficacy. Biomacromolecules.

[19] Yang C., Gao S., Dagnæs-Hansen F., Jakobsen M., Kjems J. (2017). Impact of PEG Chain Length on the Physical Properties and Bioactivity of PEGylated Chitosan/SiRNA Nanoparticles In Vitro and In Vivo. ACS Appl. Mater. Interfaces.

[20] Rheiner S., Bae Y. (2016). Increased Poly(Ethylene Glycol) Density Decreases Transfection Efficacy of SiRNA/Poly(Ethylene Imine) Complexes. AIMS Bioeng..

[21] Zhou K., Liu H., Zhang S., Huang X., Wang Y., Huang G., Sumer B.D., Gao J. (2012). Multicolored pH-Tunable and Activatable Fluorescence Nanoplatform Responsive to Physiologic PH Stimuli. J. Am. Chem. Soc..

[22] Weißpflog J., Vehlow D., Müller M., Kohn B., Scheler U., Boye S., Schwarz S. (2021). Characterization of Chitosan with Different Degree of Deacetylation and Equal Viscosity in Dissolved and Solid State–Insights by Various Complimentary Methods. Int. J. Biol. Macromol..

[23] Zhang K., Helm J., Peschel D., Gruner M., Groth T., Fischer S. (2010). NMR and FT Raman Characterisation of Regioselectively Sulfated Chitosan Regarding the Distribution of Sulfate Groups and the Degree of Substitution. Polymer.

[24] Mansur H.S., Mansur A.A.P., Curti E., De Almeida M.V. (2013). Functionalized-Chitosan/Quantum Dot Nano-Hybrids for Nanomedicine Applications: Towards Biolabeling and Biosorbing Phosphate Metabolites. J. Mater. Chem. B.

[25] Rumengan I.F.M., Suryanto E., Modaso R., Wullur S., Tallei T.E., Limbong D. (2014). Structural Characteristics of Chitin and Chitosan Isolated from the Biomass of Cultivated Rotifer, Brachionus Rotundiformis. Int. J. Fish. Aquat. Sci..

[26] Yuan Y., Chesnutt B.M., Haggard W.O., Bumgardner J.D. (2011). Deacetylation of Chitosan: Material Characterization and In Vitro Evaluation via Albumin Adsorption and Pre-Osteoblastic Cell Cultures. Materials.

[27] Oliveira F.D.P.P., Picola I.P.D., Shi Q., Barbosa H.F.G., de Oliveira Tiera V.A., Fernandes J.C., Tiera M.J. (2013). Synthesis and Evaluation of Diethylethylamine-Chitosan for Gene Delivery: Composition Effects on the In Vitro Transfection Efficiency. Nanotechnology.

[28] Foroozandeh P., Aziz A.A. (2018). Insight into Cellular Uptake and Intracellular Trafficking of Nanoparticles. Nanoscale Res. Lett..

[29] Doriti A., Brosnan S.M., Weidner S.M., Schlaad H. (2016). Synthesis of Polysarcosine from Air and Moisture Stable N-Phenoxycarbonyl-N-Methylglycine Assisted by Tertiary Amine Base. Polym. Chem..

[30] Jones C.H., Chen C.K., Ravikrishnan A., Rane S., Pfeifer B.A. (2013). Overcoming Nonviral Gene Delivery Barriers: Perspective and Future. Mol. Pharm..

[31] De Tian W., Ma Y.Q. (2012). pH-Responsive Dendrimers Interacting with Lipid Membranes. Soft Matter.

[32] Wang C., Zhao T., Li Y., Huang G., White M.A., Gao J. (2017). Investigation of Endosome and Lysosome Biology by Ultra pH-Sensitive Nanoprobes. Adv. Drug Deliv. Rev..

[33] Jiang L.Q., Wang T.Y., Webster T.J., Duan H.J., Qiu J.Y., Zhao Z.M., Yin X.X., Zheng C.L. (2017). Intracellular Disposition of Chitosan Nanoparticles in Macrophages: Intracellular Uptake, Exocytosis, and Intercellular Transport. Int. J. Nanomed..

[34] Wojnilowicz M., Glab A., Bertucci A., Caruso F., Cavalieri F. (2019). Super-Resolution Imaging of Proton Sponge-Triggered Rupture of Endosomes and Cytosolic Release of Small Interfering RNA. ACS Nano.

[35] Casey J.R., Grinstein S., Orlowski J. (2010). Sensors and Regulators of Intracellular pH. Nat. Rev. Mol. Cell Biol..

[36] Kulkarni A.D., Vanjari Y.H., Sancheti K.H., Patel H.M., Belgamwar V.S., Surana S.J., Pardeshi C.V. (2016). Polyelectrolyte Complexes: Mechanisms, Critical Experimental Aspects, and Applications. Artif. Cells Nanomed. Biotechnol..

[37] Kim Y.H., Lee K., Li S. (2021). Nucleic Acids Based Polyelectrolyte Complexes: Their Complexation Mechanism, Morphology, and Stability. Chem. Mater..

[38] Agirre M., Zarate J., Ojeda E., Puras G., Desbrieres J., Pedraz J.L. (2014). Low Molecular Weight Chitosan (LMWC)-Based Polyplexes for pDNA Delivery: From Bench to Bedside. Polymers.

[39] Nel A.E., Mädler L., Velegol D., Xia T., Hoek E.M.V., Somasundaran P., Klaessig F., Castranova V., Thompson M. (2009). Understanding Biophysicochemical Interactions at the Nano-Bio Interface. Nat. Mater..

[40] Tiera M.J., Shi Q., Winnik F.M., Fernandes J.C. (2011). Polycation-Based Gene Therapy: Current Knowledge and New Perspectives. Curr. Gene Ther..

[41] Picola I.P.D., Busson K.A.N., Casé A.H., Nasário F.D., Tiera V.A.D.O., Taboga S.R., Neto J.R., Tiera M.J. (2013). Effect of Ionic Strength Solution on the Stability of Chitosan-DNA Nanoparticles. J. Exp. Nanosci..

[42] Mazumdar S., Chitkara D., Mittal A. (2021). Exploration and Insights into the Cellular Internalization and Intracellular Fate of Amphiphilic Polymeric Nanocarriers. Acta Pharm. Sin. B.

[43] Cabral H., Miyata K., Osada K., Kataoka K. (2018). Block Copolymer Micelles in Nanomedicine Applications. Chem. Rev..

[44] Hühn D., Kantner K., Geidel C., Brandholt S., De Cock I., Soenen S.J.H., Riveragil P., Montenegro J.M., Braeckmans K., Müllen K. (2013). Polymer-Coated Nanoparticles Interacting with Proteins and Cells: Focusing on the Sign of the Net Charge. ACS Nano.

[45] Giacomelli F.C., Stepánek P., Giacomelli C., Schmidt V., Jäger E., Jäger A., Ulbrich K. (2011). pH-Triggered Block Copolymer Micelles Based on a PH-Responsive PDPA (Poly [2-(Diisopropylamino)Ethyl Methacrylate]) Inner Core and a PEO (Poly(Ethylene Oxide)) Outer Shell as a Potential Tool for the Cancer Therapy. Soft Matter.

[46] Kun R., Szekeres M., Dékány I. (2009). Isothermal Titration Calorimetric Studies of the pH Induced Conformational Changes of Bovine Serum Albumin. J. Therm. Anal. Calorim..

[47] Li Y., Zeng X., Wang S., Sun Y., Wang Z., Fan J., Song P., Ju D. (2015). Inhibition of Autophagy Protects against PAMAM Dendrimers-Induced Hepatotoxicity. Nanotoxicology.

[48] Xu Z., Shen G., Xia X., Zhao X., Zhang P., Wu H., Guo Q., Qian Z., Wei Y., Liang S. (2011). Comparisons of Three Polyethyleneimine-Derived Nanoparticles as a Gene Therapy Delivery System for Renal Cell Carcinoma. J. Transl. Med..

[49] Liu S., Gao Y., Zhou D., Zeng M., Alshehri F., Newland B., Lyu J., O’Keeffe-Ahern J., Greiser U., Guo T. (2019). Highly Branched Poly(β-Amino Ester) Delivery of Minicircle DNA for Transfection of Neurodegenerative Disease Related Cells. Nat. Commun..

[50] Raik S.V., Andranovitš S., Petrova V.A., Xu Y., Lam J.K.W., Morris G.A., Brodskaia A.V., Casettari L., Kritchenkov A.S., Skorik Y.A. (2018). Comparative Study of Diethylaminoethyl-Chitosan and Methylglycol-Chitosan as Potential Non-Viral Vectors for Gene Therapy. Polymers.

[51] BCRJ: Banco de Células do Rio de Janeiro (RAW 264.7|APABCAM). https://bcrj.org.br/celula/0212.

[52] BCRJ: Banco de Células do Rio de Janeiro (HeLa/GFP|APABCAM). https://bcrj.org.br/celula/HELA-GFP-CERVIX-ADENOCARCINOMA.

[53] ATCC® (NIH/3T3). https://www.atcc.org/products/crl-1658.

[54] Tiera M.J., Qiu X.P., Bechaouch S., Shi Q., Fernandes J.C., Winnik F.M. (2006). Synthesis and Characterization of Phosphorylcholine-Substituted Chitosans Soluble in Physiological pH Conditions. Biomacromolecules.

[55] Ma O., Lavertu M., Sun J., Nguyen S., Buschmann M.D., Winnik F.M., Hoemann C.D. (2008). Precise Derivatization of Structurally Distinct Chitosans with Rhodamine B Isothiocyanate. Carbohydr. Polym..

[56] Casé A.H., Picola I.P.D., Zaniquelli M.E.D., Fernandes J.C., Taboga S.R., Winnik F.M., Tiera M.J. (2009). Physicochemical Characterization of Nanoparticles Formed between DNA and Phosphorylcholine Substituted Chitosans. J. Colloid Interface Sci..

[57] Dragicevic N., Maibach H.I. (2016). Percutaneous Penetration Enhancers Chemical Methods in Penetration Enhancement: Nanocarriers.

