# Supplementary material for: Impact of Degree of Ionization and PEGylation on the Stability of Nanoparticles of Chitosan Derivatives at Physiological Conditions
Source: Mar Drugs. 2022 Jul 25;20(8):476. doi: 10.3390/md20080476 (PMC9330794; doi:10.3390/md20080476)
Supplement: Supplementary file 1 [file marinedrugs-20-00476-s001.zip › marinedrugs-1809915-supplementary.pdf]

Supplementary Information

Impact of degree of ionization and PEGylation on the stability of nanoparticles of chitosan derivatives at physiological conditions

André Miguel Martinez Junior[a], Aline Margarete Furuyama[a], Grazieli Olinda Martins[a], Vera Aparecida de Oliveira[a], Mohamed Benderdour[b], Julio Cesar Fernandes[b], Marcio José Tiera[a]*

[a] Department of Chemistry and Environmental Sciences, IBILCE, São Paulo State University – UNESP, São José do Rio Preto, São Paulo, Brazil.

[b] Orthopedic Research Laboratory, Hôpital du Sacré-Coeur de Montréal, Université de Montréal-Canada.

Corresponding Author

* Marcio José Tiera, Associate Professor, Department of Chemistry and Environmental Sciences,

IBILCE, UNESP, R. Cristóvão Colombo, 2265, 15054-000 São José do Rio Preto, SP, Brazil.

Tel. 55-17-32212359. Fax 55-17-32212356.

Email address: marcio.tiera@ibilce.unesp.br

^{1}H and ^{13}C NMR spectra

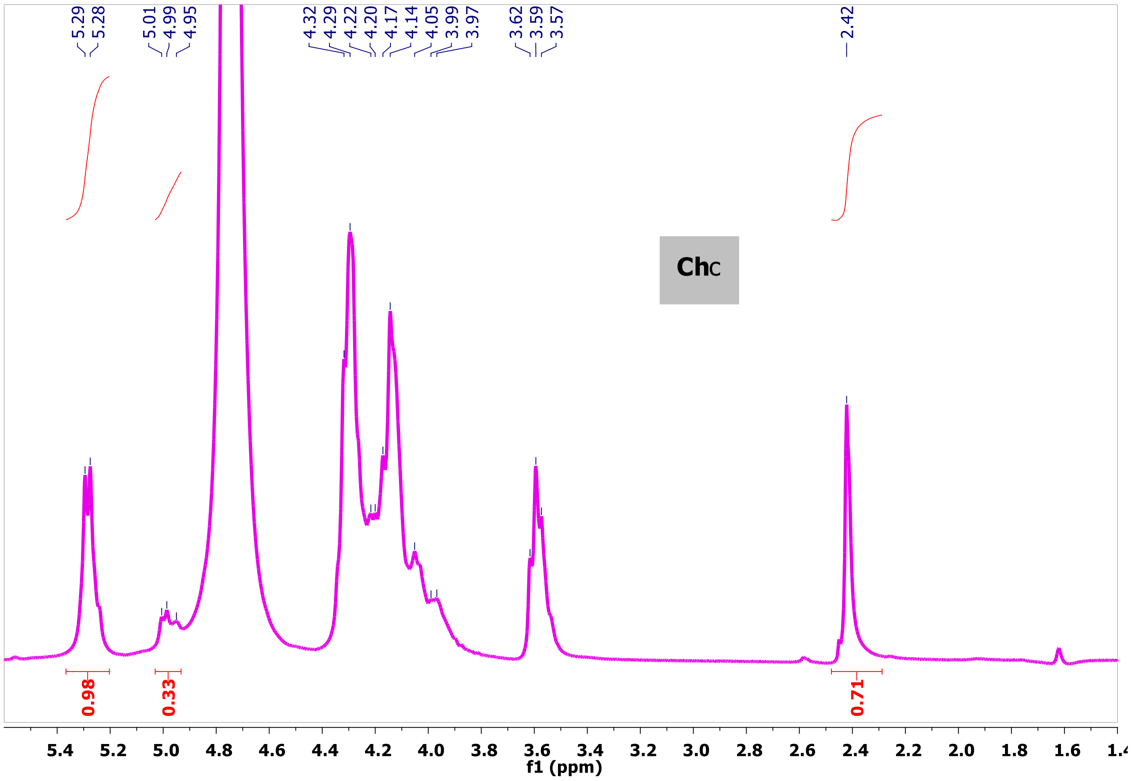

Figure S1. ^{1}H NMR spectrum of Ch_c (commercially obtained chitosan).

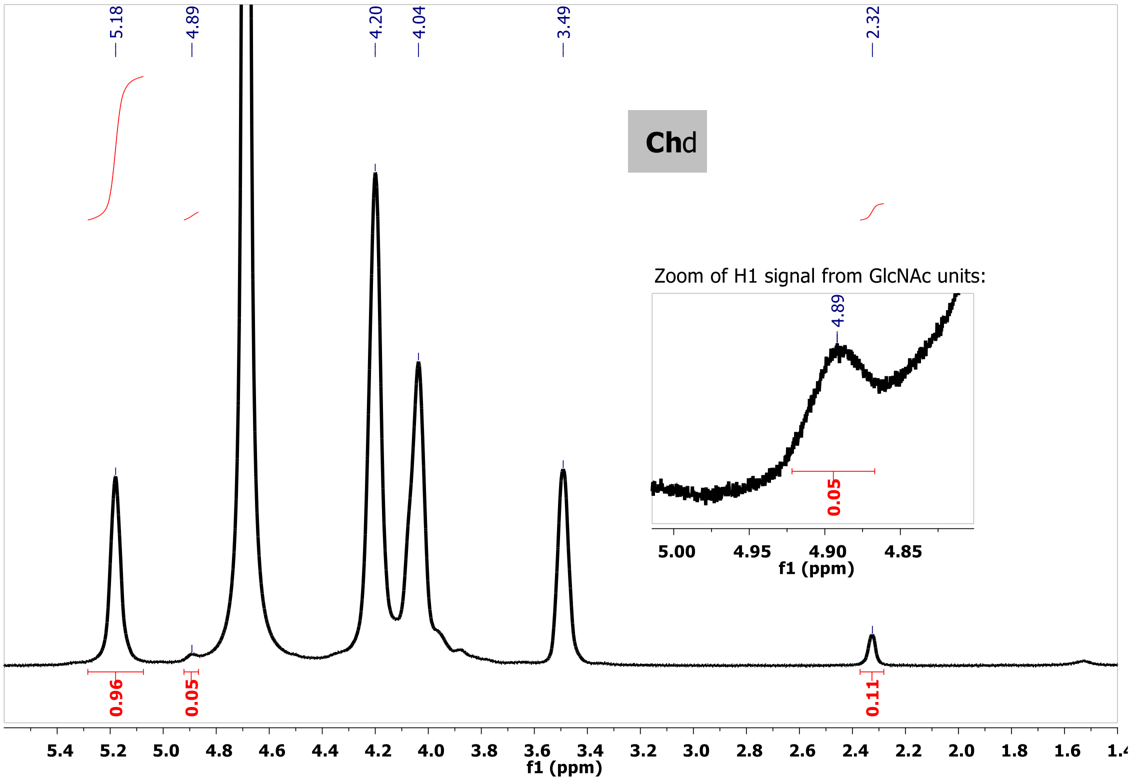

Figure S2. ^{1}H NMR spectrum of Ch_d (deacetylated chitosan).

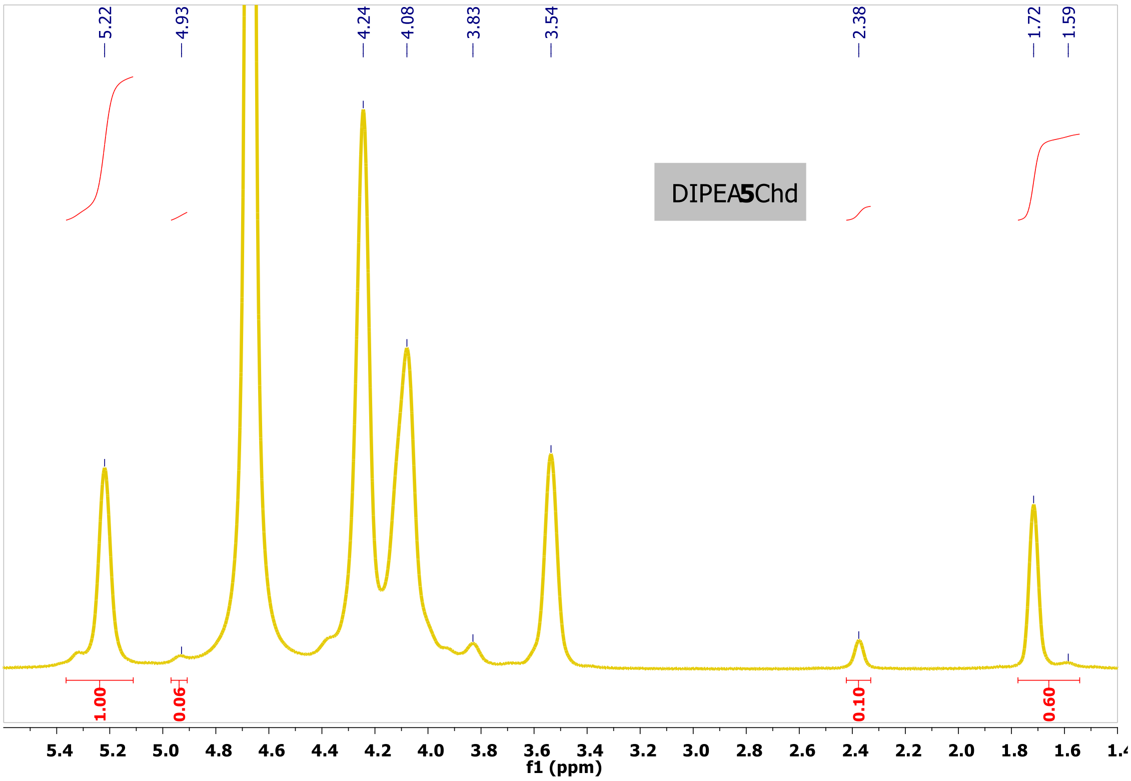

Figure S3. ^{1}H NMR spectrum of DIPEA$_5$Ch$_d$ derivative.

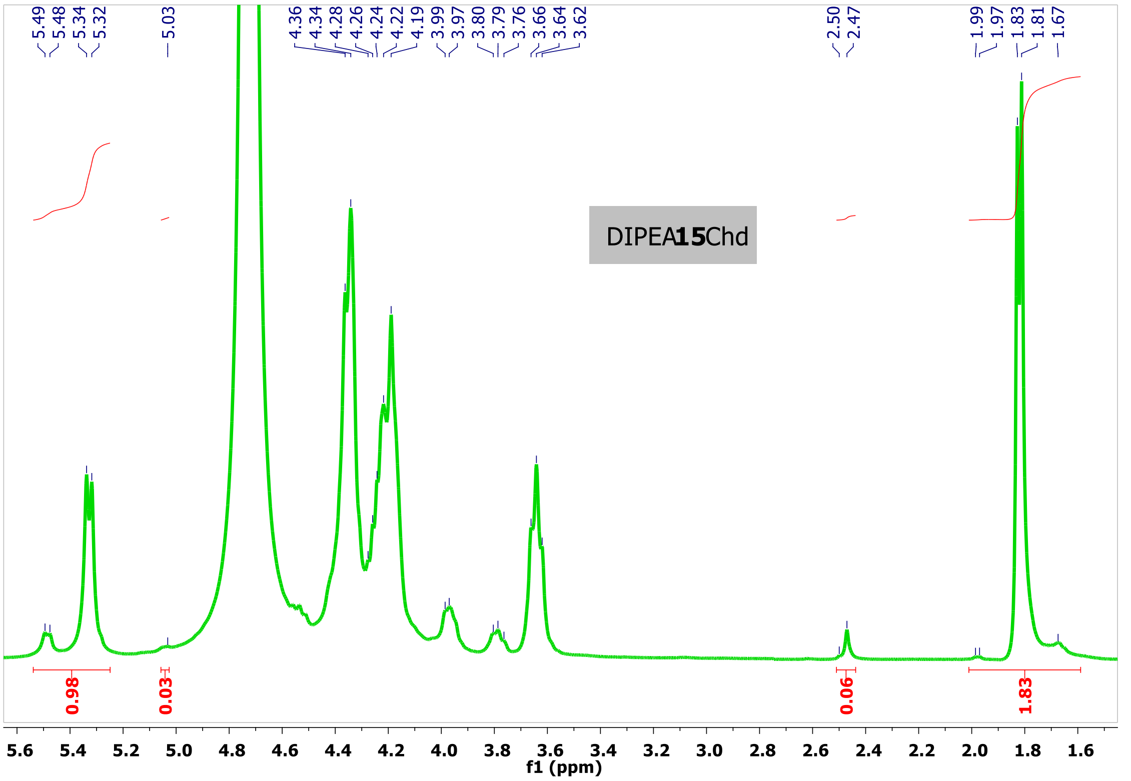

Figure S4. ^{1}H NMR spectrum of DIPEA$_{15}$Ch$_d$ derivative.

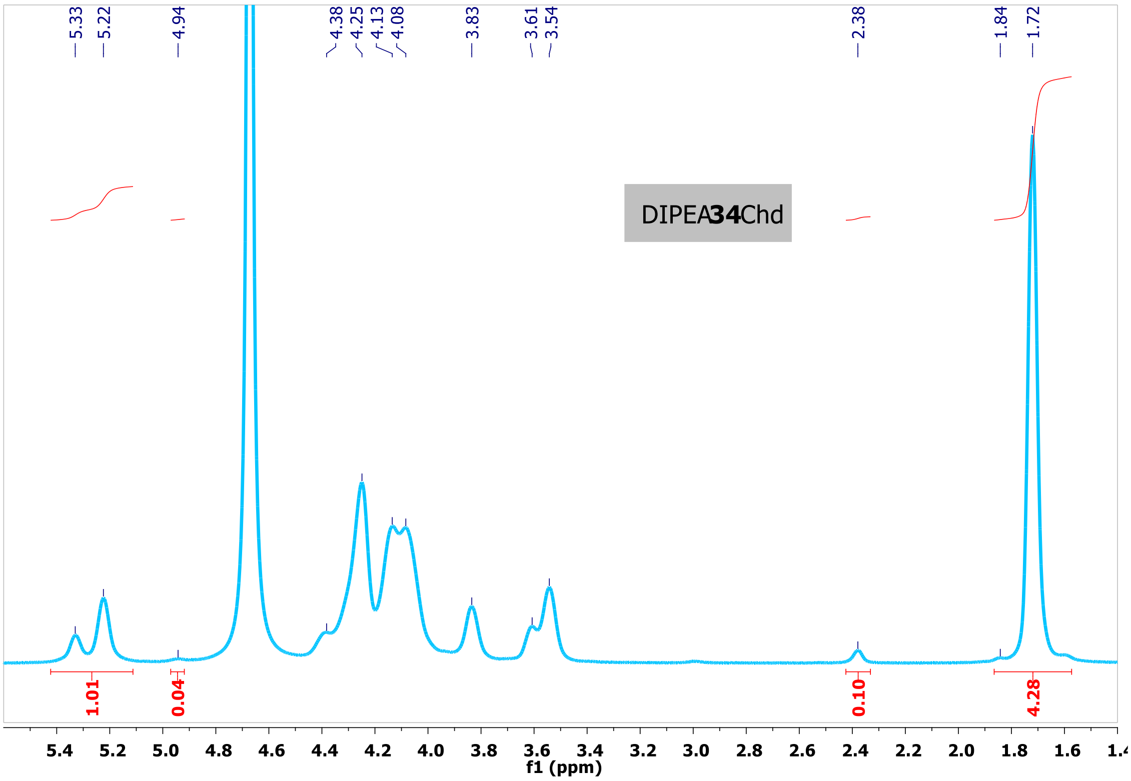

Figure S5. ^{1}H NMR spectrum of DIPEA$_{34}$Ch$_{d}$ derivative.

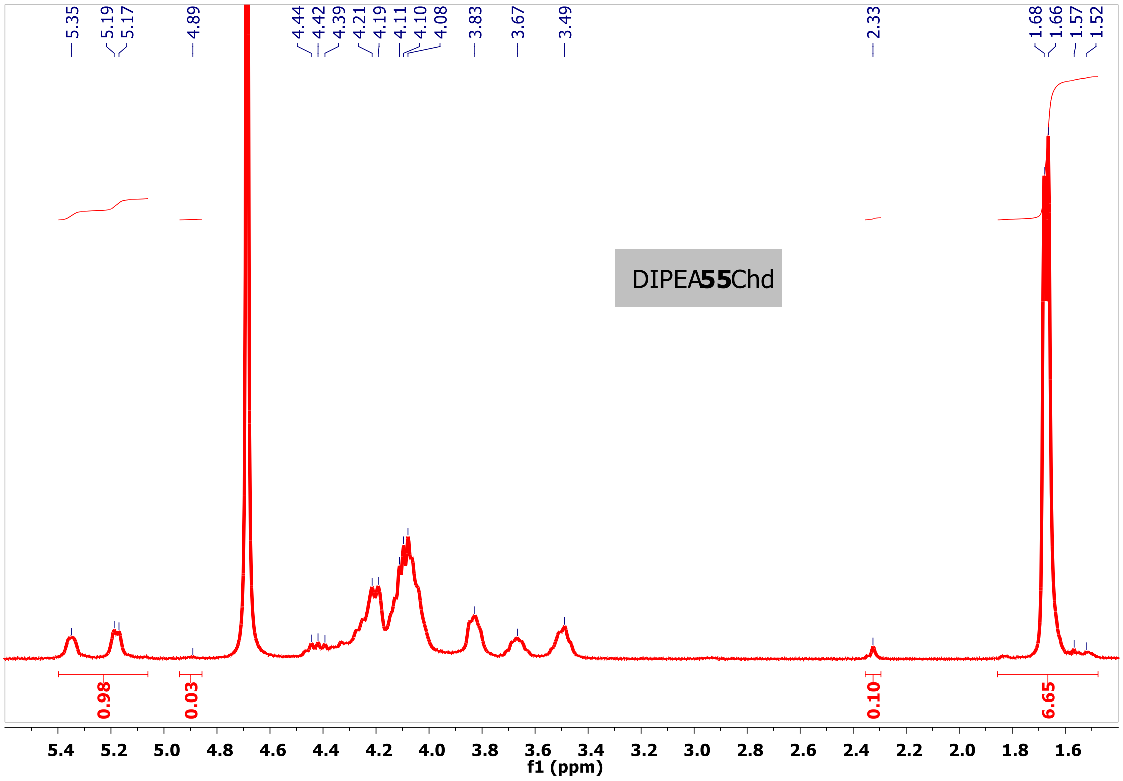

Figure S6. ^{1}H NMR spectrum of DIPEA$_{55}$Ch$_{d}$ derivative.

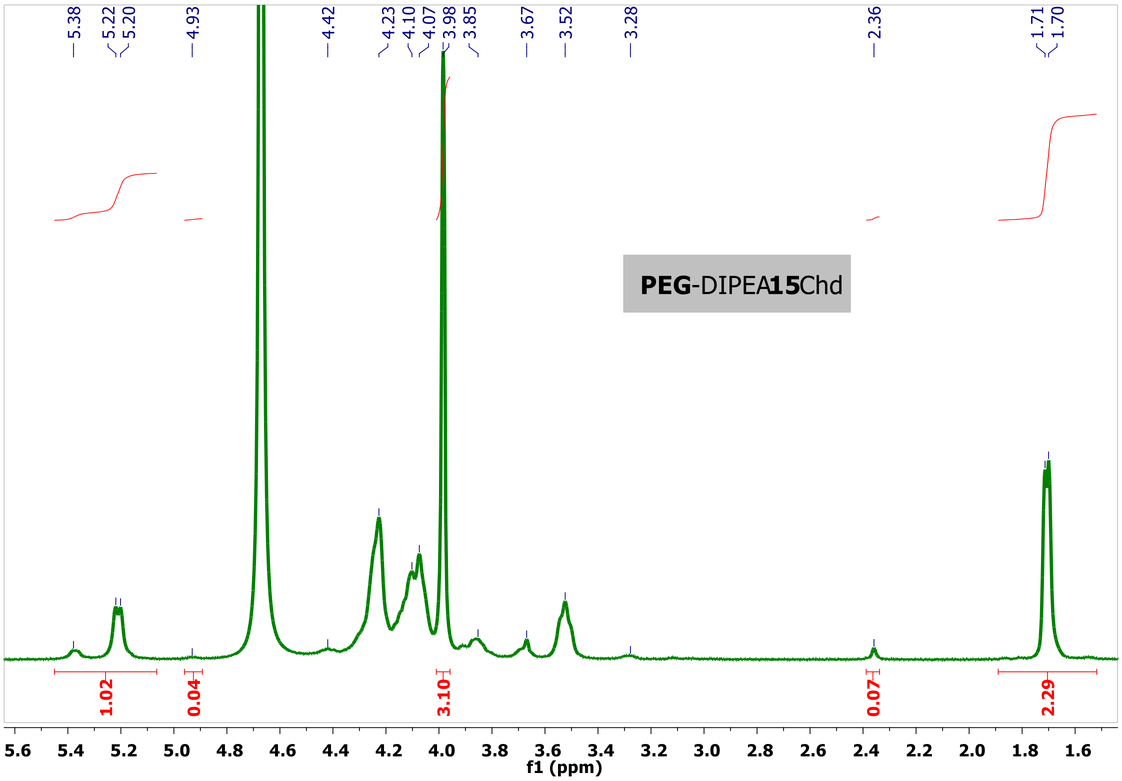

Figure S7. ^{1}H NMR spectrum of PEG-DIPEA$_{15}$Ch$_d$ derivative.

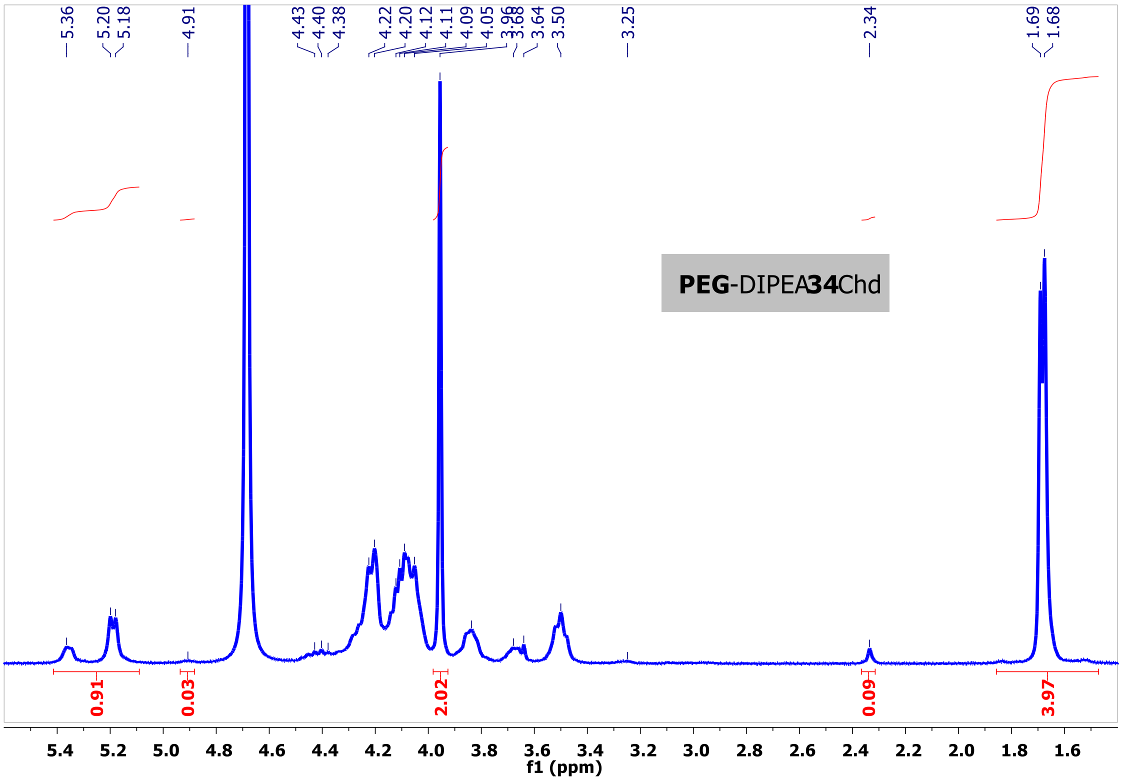

Figure S8. ^{1}H NMR spectrum of PEG-DIPEA$_{34}$Ch$_d$ derivative.

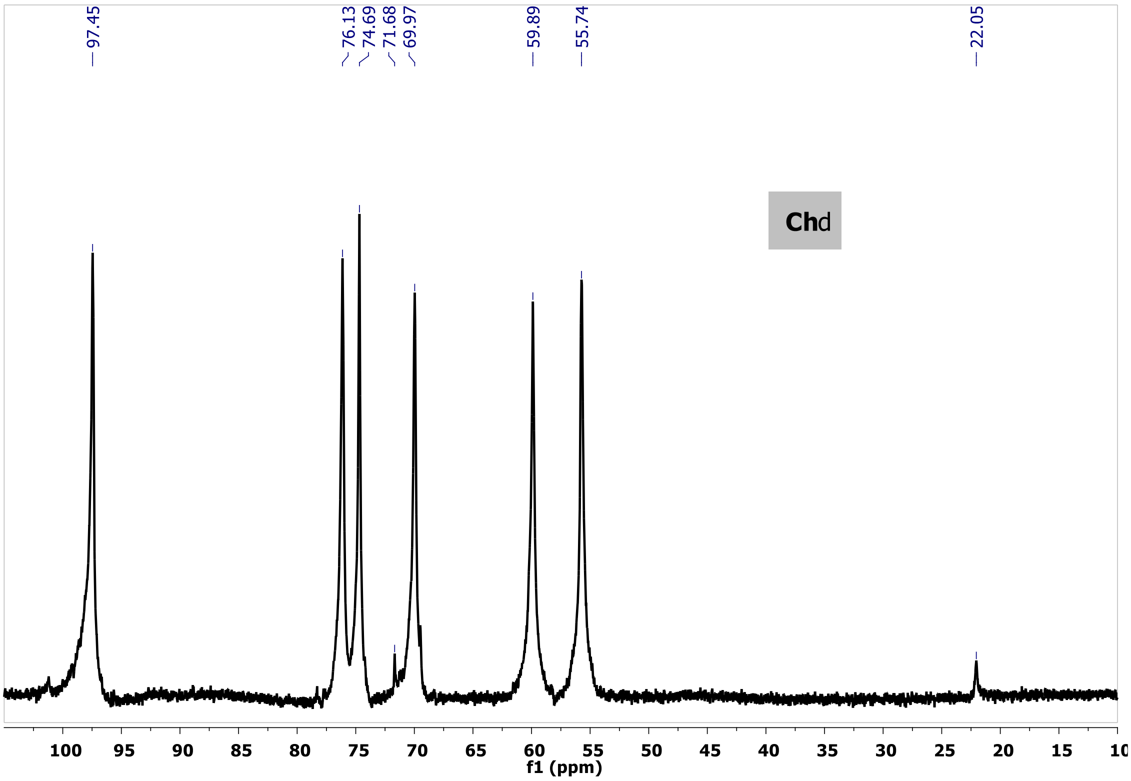

Figure S9. ^{13}C NMR spectrum of Ch_d (deacetylated chitosan).

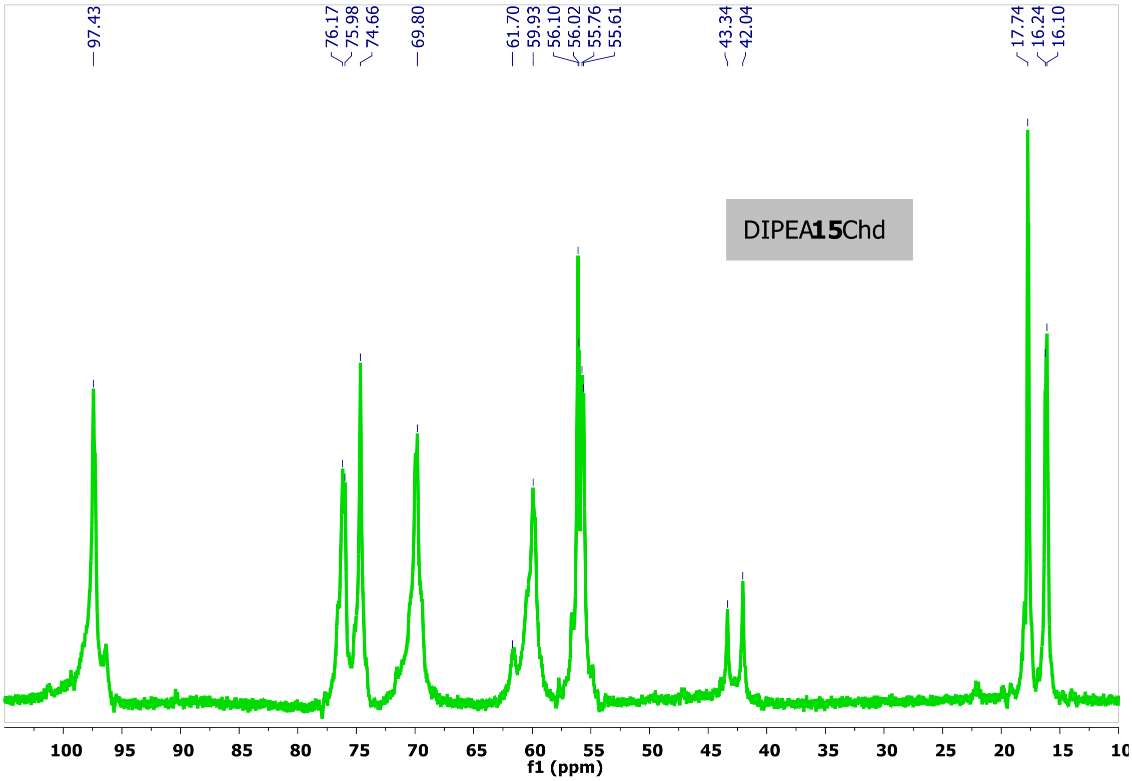

Figure S10. ^{13}C NMR spectrum of $DIPEA_{15}Ch_d$ derivative.

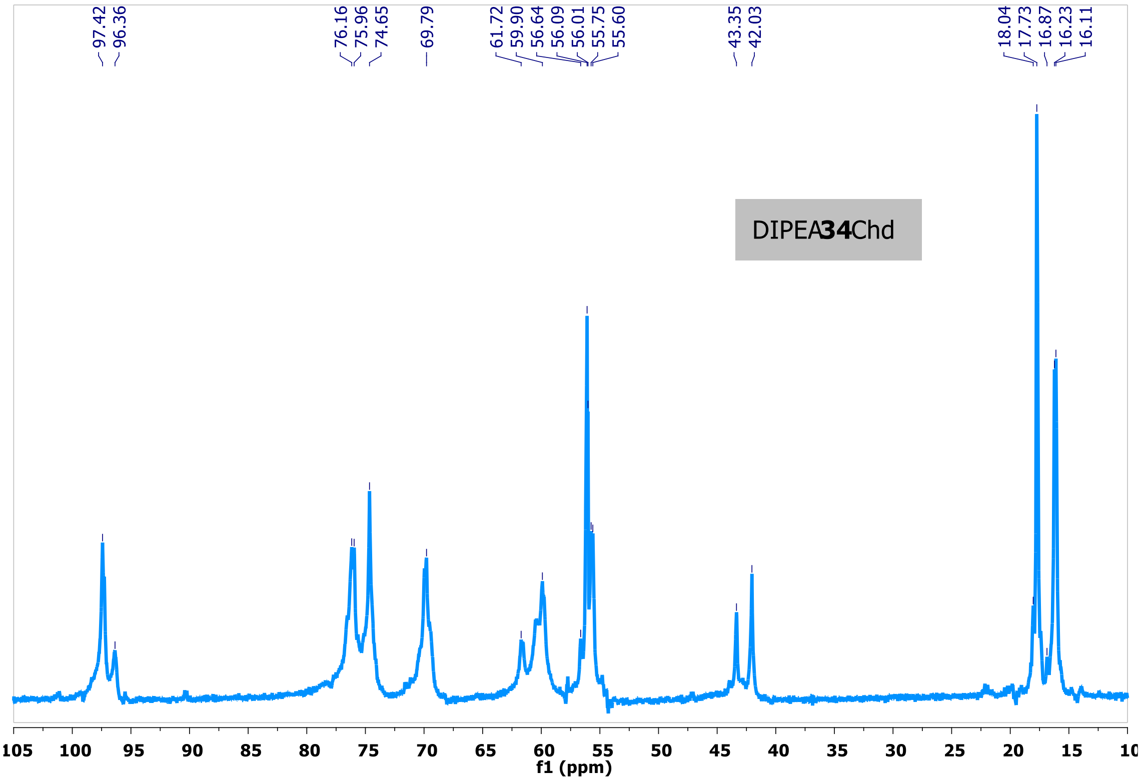

Figure S11. ^{13}C NMR spectrum of $DIPEA_{34}Ch_d$ derivative.

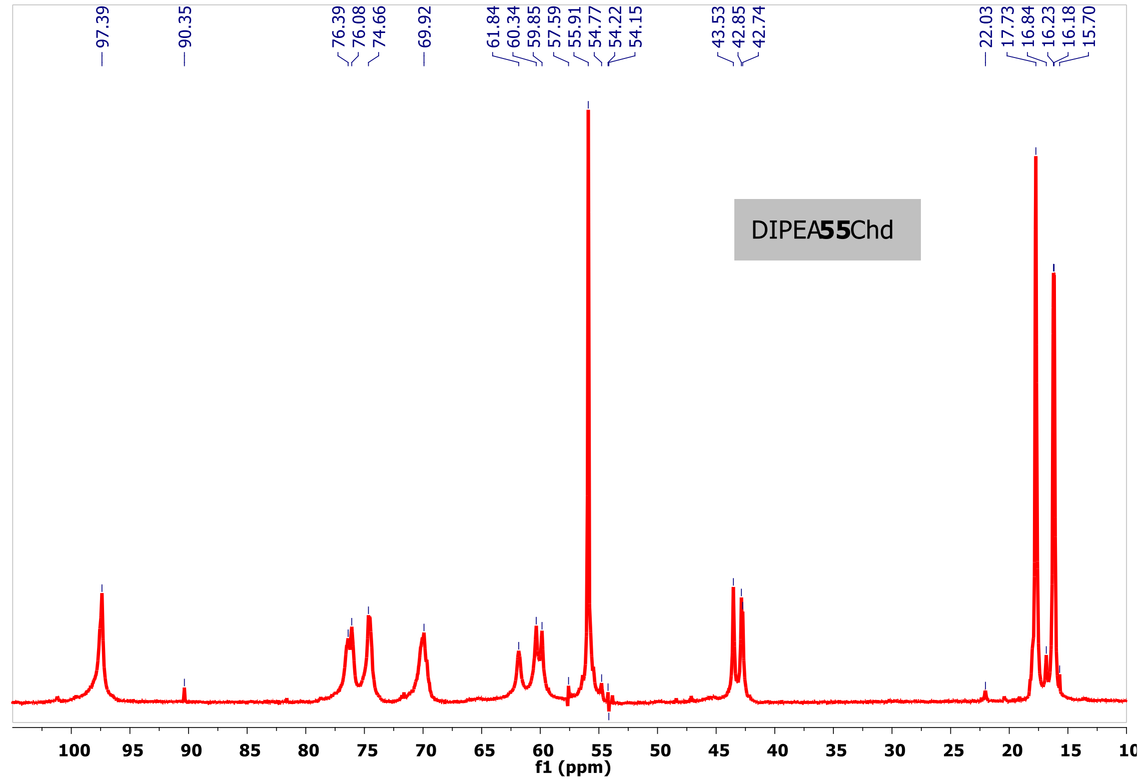

Figure S12. ^{13}C NMR spectrum of $DIPEA_{55}Ch_d$ derivative.

Degree of labeling by rhodamine isothiocyanate (RITC)

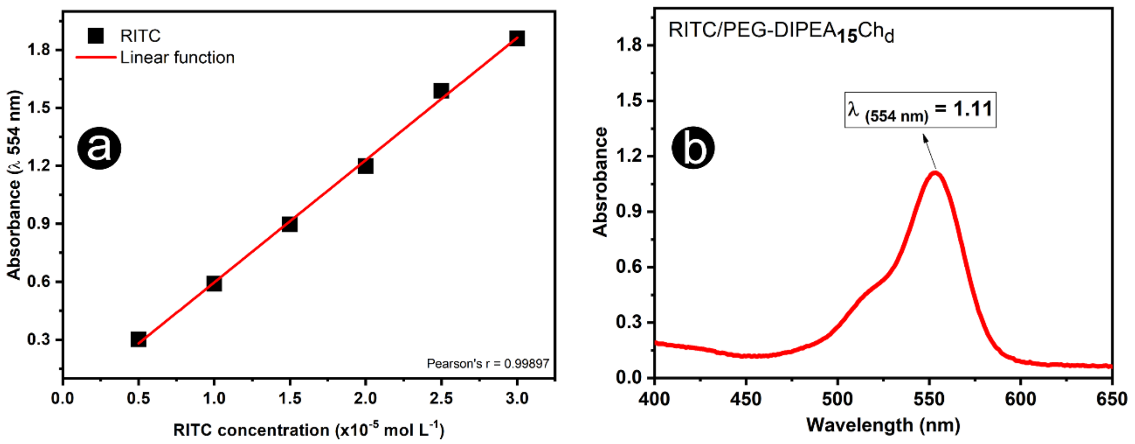

Figure S13. (a) Analytical curve of Rhodamine B isothiocyanate (RITC) in acetic acid - sodium acetate buffer pH 4.5/methanol (50:50 v/v) and (b) UV-visible absorbance spectrum of RITC/PEG-DIPEA15Chd used to determinate the DS_{RITC}.

The absorbance (Figure S13) at 554 nm (Abs_{sample}), the analytical curve and the Equation (S1) were used to determine the concentration of RITC in the labeled sample (C_{RITC}). Once the concentration of RITC was known, the degree of substitution by rhodamine (DS_{RITC}) was determined using the Equation S2. Where, n_{RITC} is the number of moles of RITC (based on C_{RITC}) and m_{pol} is the mass (g) of RITC/PEG-DIPEA$_{15}$Ch$_{d}$ in the polymeric solution. 536.1 and 216 refer to the molecular weight (g mol^{-1}) of RITC and the mean molecular weight of repetitive units of PEG-DIPEA$_{15}$Ch$_{d}$, respectively. The DS_{RITC} of RITC/PEG-DIPEA$_{15}$Ch$_{d}$ was 0.6 moles %.

$$C_{RITC} = \frac{Abs_{sample}+0,036}{63309} \quad \text{(S1)}$$

$$DS_{RITC} = \frac{n_{RITC}}{\frac{(m_{pol}-n_{RITC}\times 536,1)}{216}} \times 100 \quad \text{(S2)}$$

Gel permeation chromatography (GPC) data

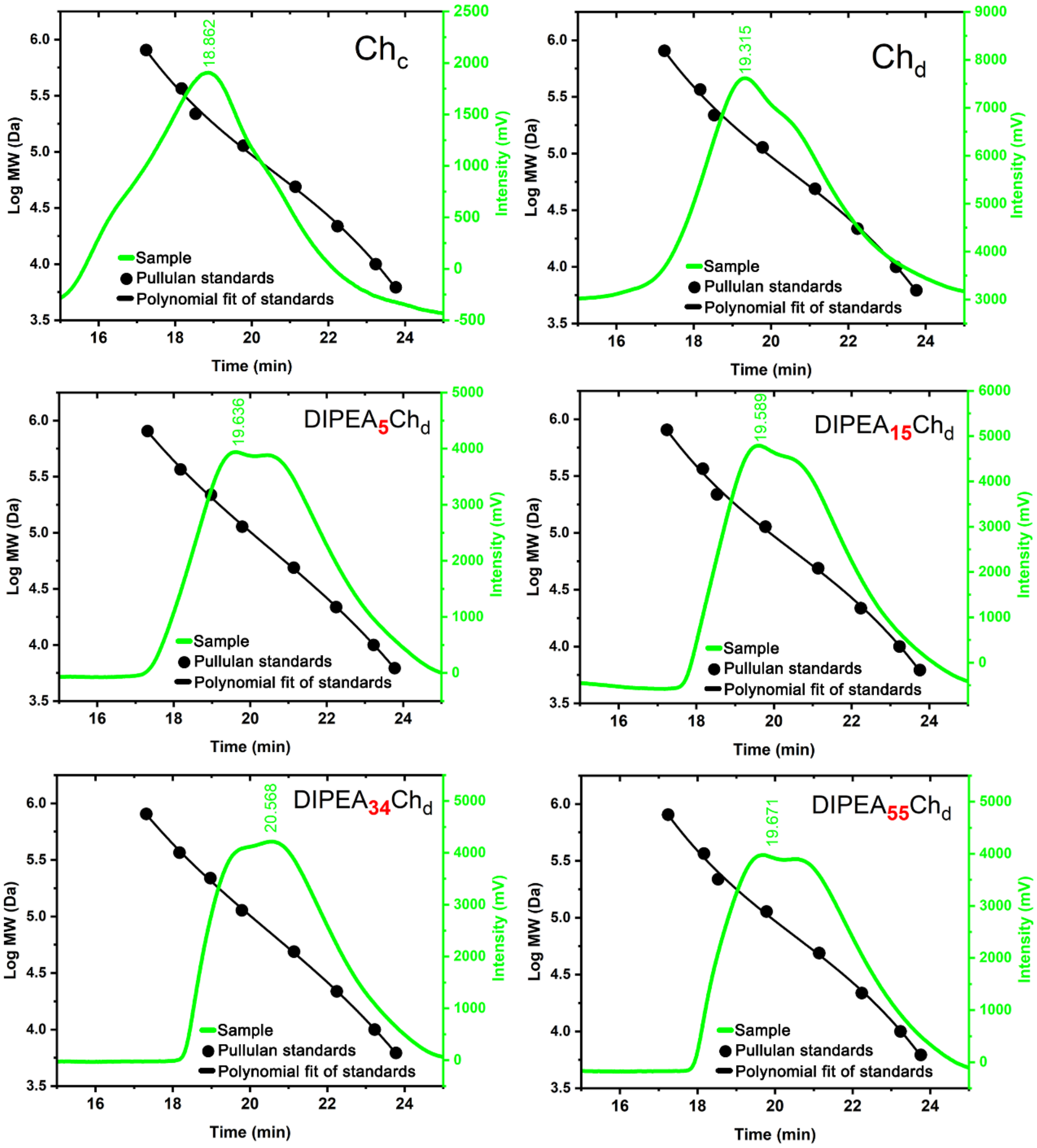

Figure S14. GPC chromatograms of chitosan and its derivatives. The analytical curve was performed using pullulan standards with molecular weight in the range of 6.2 to 805 kDa.

Conductometric and potentiometric titrations data

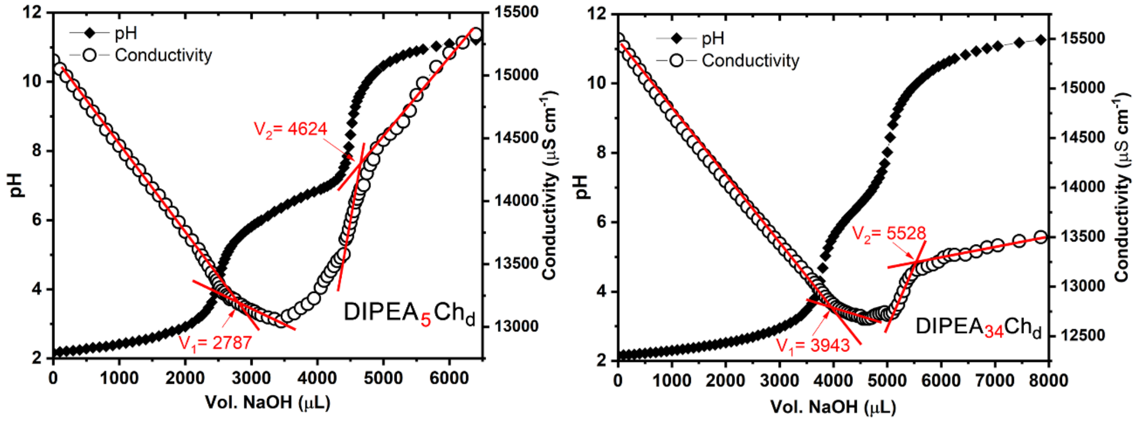

Figure S15. Conductometric and potentiometric titrations of polymeric solutions with NaOH 0.077 mol L^{-1}. V_1 and V_2 refer to the volumes of NaOH (μL) to consume the excess of HCl and to deprotonate the amine groups from polymer, respectively.

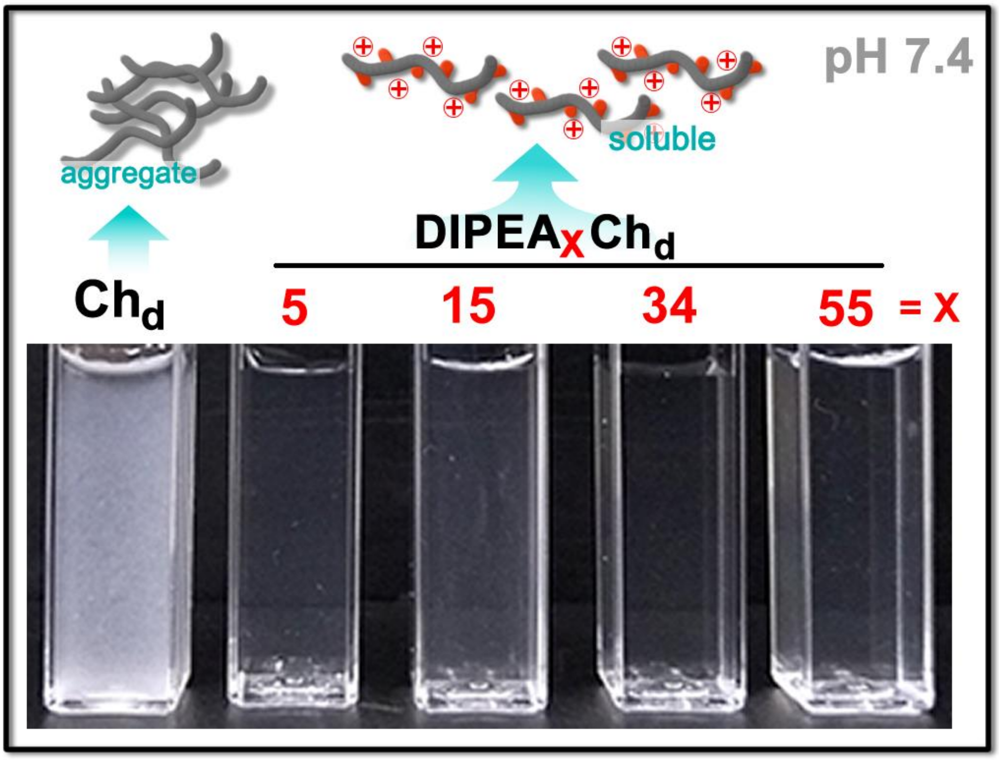

Figure S16. Solubility of Chitosan and its DIPEA derivatives at pH 7.4 and ionic strength of 150 mmol L^{-1}.

FTIR spectra

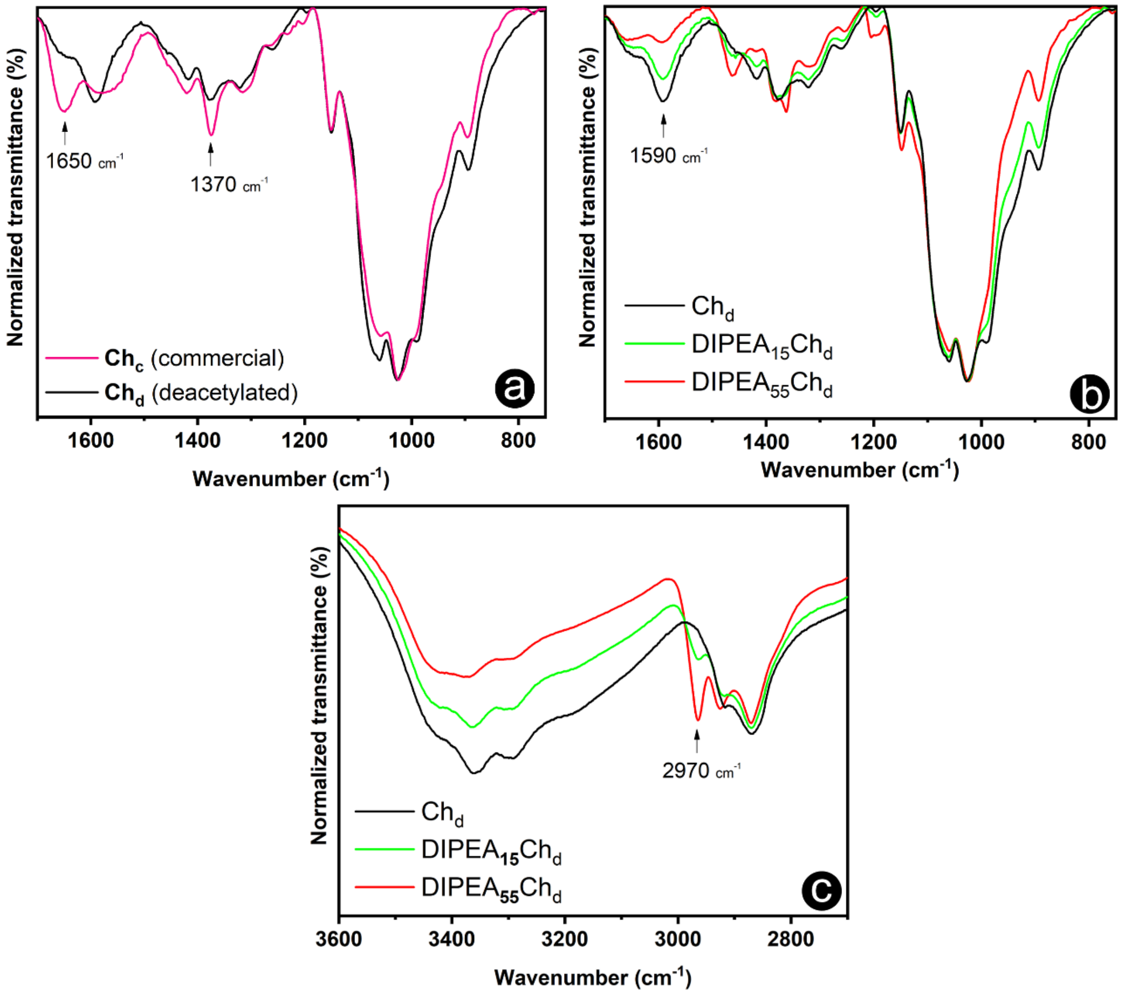

Figure S17. Magnification of selected bands from FTIR spectra of (a) commercial and deacetylated chitosan, and (b, c) DIPEA-derivatives.

Dynamic Light Scattering (DLS) of Pegylated chitosan nanoparticles

The Ch_d was PEGylated following the same procedure described for DIPEA-derivatives. Briefly, 150 mg of Ch_d was solubilized in 7.5 mL of HAc (0.03 mol L^{-1}) and diluted with PBS at a final concentration of 10 g L^{-1}. The pH of the solution was set to 7.4 by NaOH (5 mol L^{-1}) and SPDP (5.45 mg in 1 mL of DMSO) was dropped into solution under magnetic stirring. After 3 hours, PEG-SH (35 mg in 1 mL of PBS) was added to the reaction mixture and heated to 70 °C and kept under magnetic stirring for 16 hours. Finally, the PEGylated derivative (PEG-Ch_d) was purified by dialysis and recovered by lyophilization. The PEG-SH/NH_2 ratio was 0.02 and the PEG-SH/SPDP ratio was 1.0. The degree of substitution for PEG-Ch_d was 1.6% (*mol/mol*), according to ^{1}H NMR analyses. The siRNA anti-TNFα–PEGCh_d nanoparticles were formulated as described in the section 3.3.1 and the obtained light scattering data are shown in Figure S18.

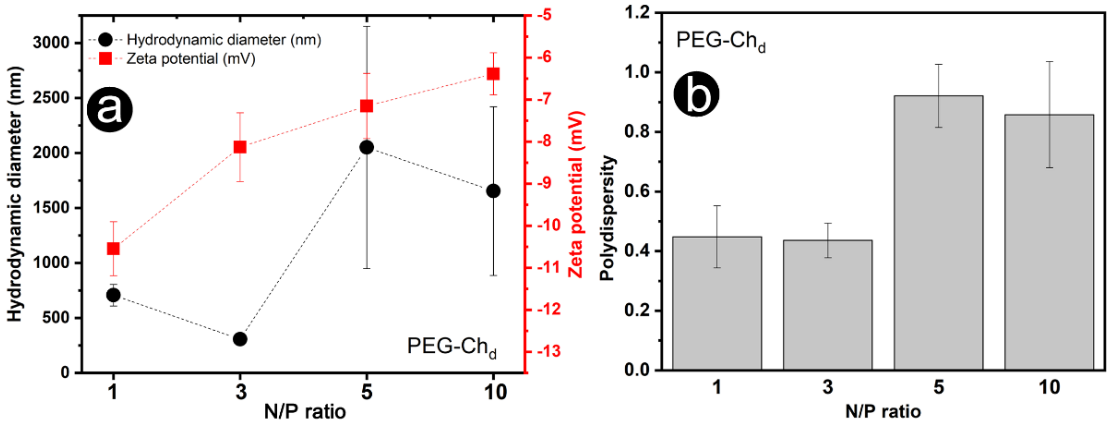

Figure S18. (a) hydrodynamic diameter, Zeta potential and (b) polydispersity of polyplexes formed with siRNA anti-TNFα–PEGCh_d (pH 7.4, I = 150 mmol L^{-1})

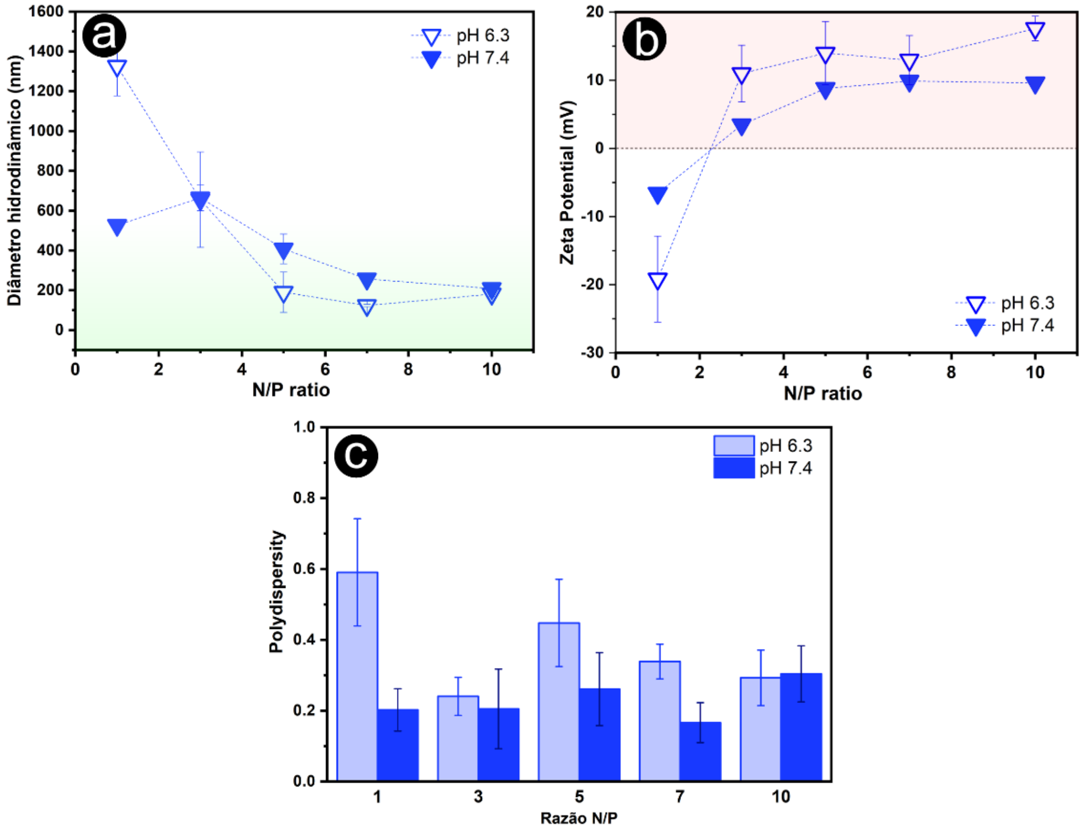

Figure S19. (a) hydrodynamic diameter (b) Zeta potential and (c) size polydispersity of nanoparticles formed by $DIPEA_{34}Ch_d$ (and siRNA anti-TNFα) at different N/P ratios at pH 6.3 (I = 50 mmol L^{-1}; Phosphate = 25 mmol L^{-1}) and 7.4 (I = 150 mmol L^{-1}, Phosphate 50 mmol L^{-1}).

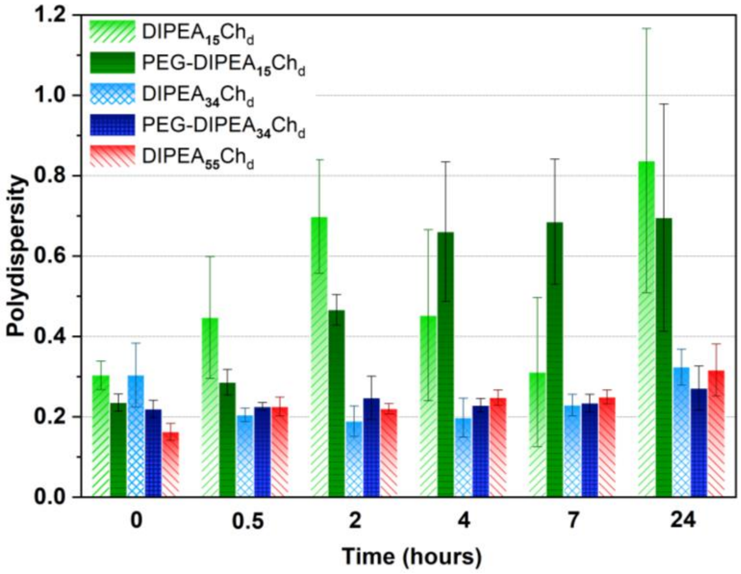

Figure S20. Polydispersity of nanoparticles as a function of time. The nanoparticles were formulated in PBS at N/P ratio 10, pH 7.4 and I = 150 mmol L^{-1}.

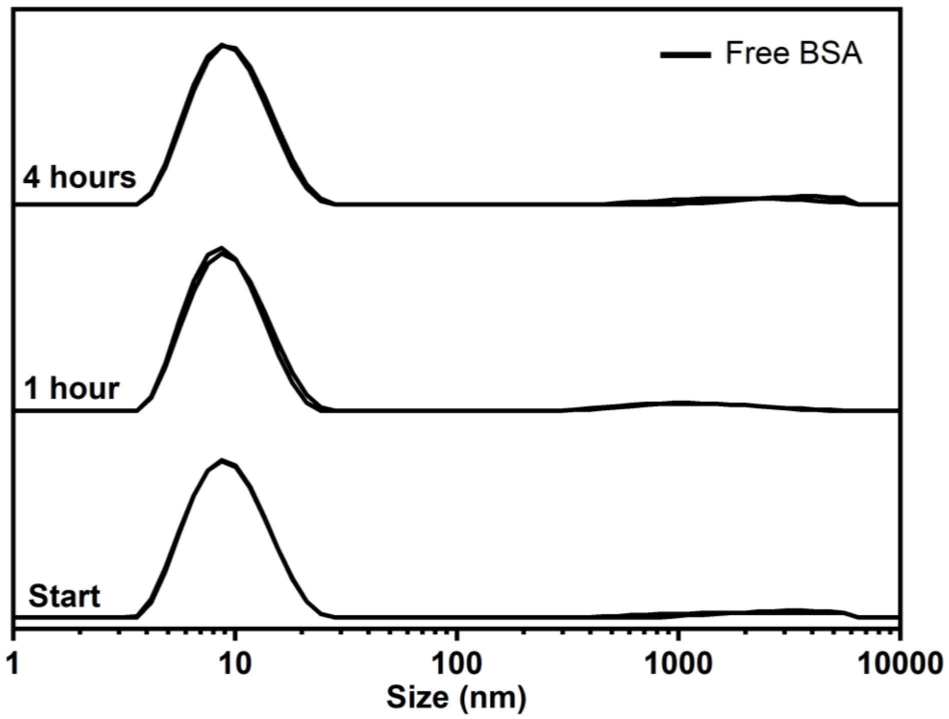

Figure S21. Size distribution of free albumin (BSA) in phosphate buffer, pH 7.4 and I = 150 mmol L^{-1} (n = 2).

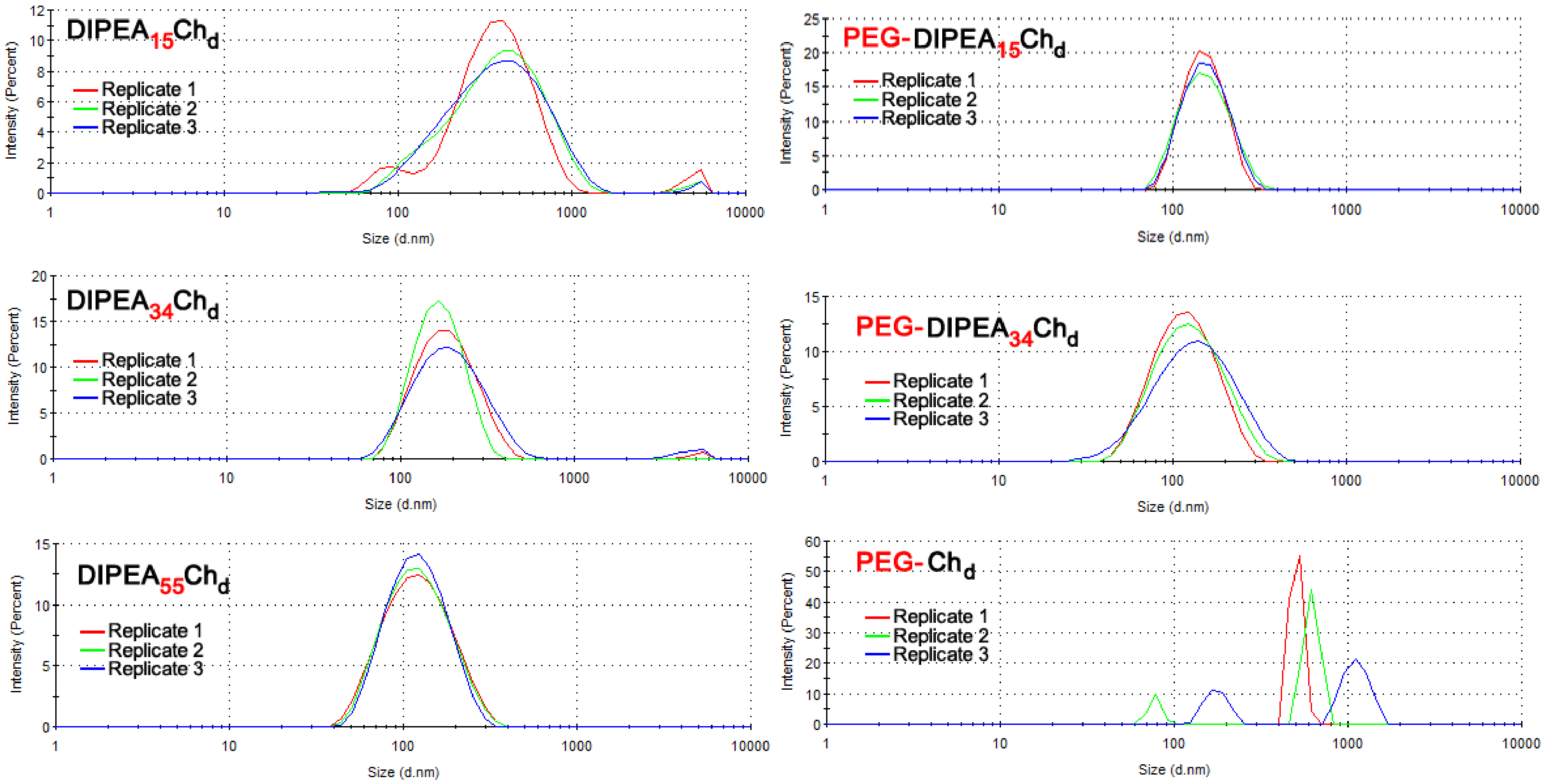

Figure S22. Figure S22. Size distribution curves of nanoparticles formulated at N/P ratio 10 (pH 7.4 and I = 150 mmol L^{-1} . (n = 3).

GFP knockdown

The estimated efficiency of GFP knockdown in HeLa-GFP cells treated with siRNA anti-GFP-nanoparticles was made using ImageJ software (version 1.53k). The image obtained by confocal microscopy (DAPI and GFP channels) were converted to an 8-bit image (black and white) and adjusted by the auto threshold (v1.17) following the Huang2 method (white objects on black background). Then, the images were analyzed (analyze particles) with no set values (size: 0-Infinity; circulatory: 0.0-1.0) for quantification of white areas. For the data treatment, the total white area of the GFP images was normalized by the total white area of the DAPI image (Figure S23). Considering the GFP/DAPI area ratio of non-treated cells as 100% of GFP expression, the cells treated by PEG-$DIPEA_{15}Ch_{d}$ and Lipofectamine 2000 presented 73% and 71% of GFP expression, respectively.

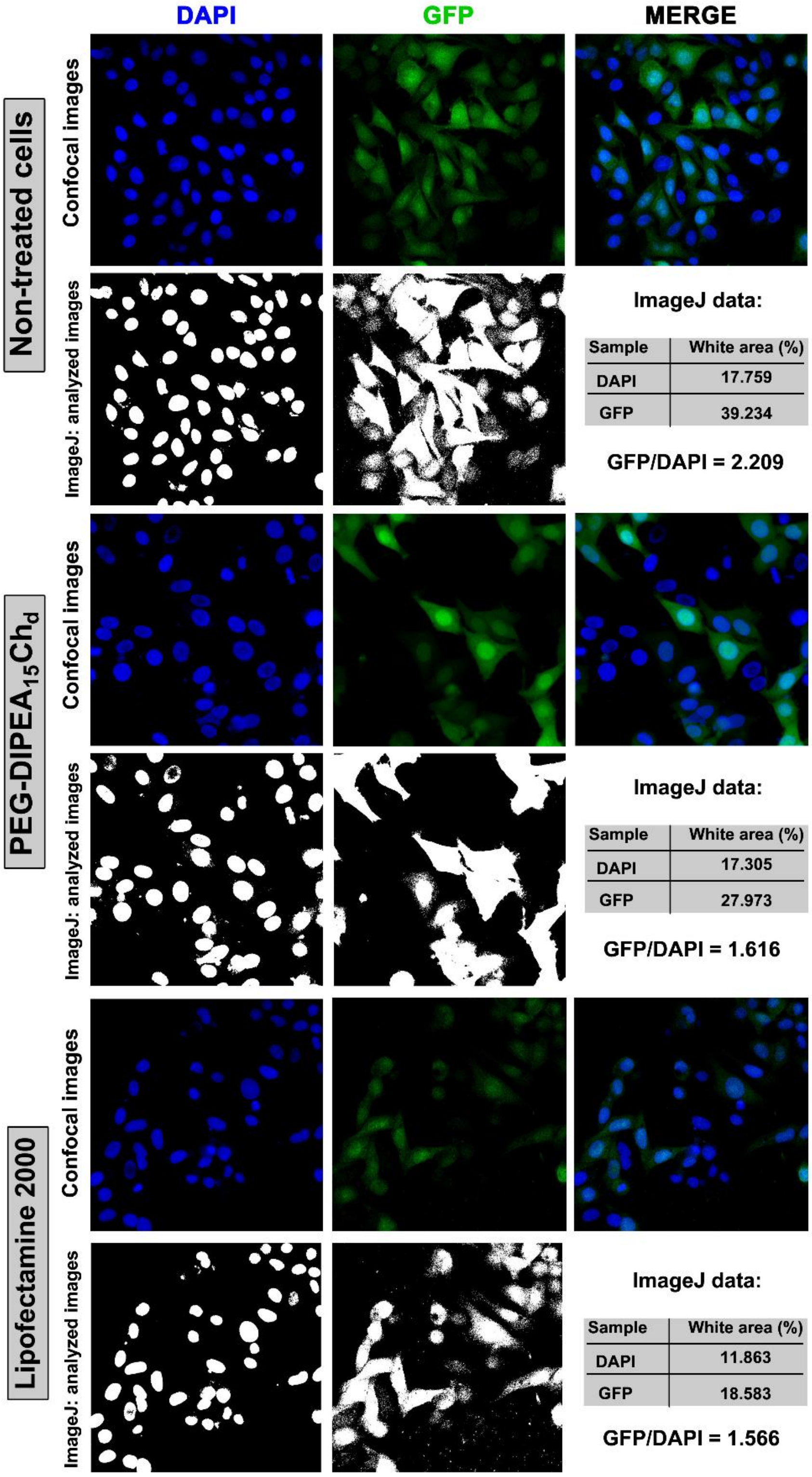

Figure S23. (colorful) Confocal microscopy images of non-treated and treated HeLa-GFP cells with siRNA anti-GFP- nanoparticles of PEG-DIPEA$_{15}$Ch$_d$ and Lipofectamine (black-and-white). Images analyzed by ImageJ software for the white area measurement.
